# Precision epigenetic therapies in oncology

**DOI:** 10.1007/s10555-025-10288-w

**Published:** 2025-09-19

**Authors:** Arundhati Chaudhary, Kayleigh J. A. Orchard, Francesca Salani, Theodora Partsou, Mark Eccleston, Guido Bocci, Antoine Italiano, Francesco Crea

**Affiliations:** 1 Cancer Research Group, School of Life, Health and Chemical Sciences, The Open University, Walton Hall, Milton Keynes, MK7 6AA UK; 2 American College of Thessaloniki, Vasiliou Sevenidi 17, Pilea, 55535 Greece; 3 Sant’ Anna School of Advanced Studies, Institute of Life Sciences, Piazza Martiri della Liberta 33, Pisa, 56124 Italy; 4 Department of Translational Research and New Technologies in Medicine and Surgery, School of Medicine, University of Pisa, Pisa, Italy; 5 Faculty of Medicine Institut Bergonié, Université of Bordeaux, Bordeaux, France; 6 ValiRx plc, MediCity D6 Thane Road, Nottingham, NG90 6BH UK

**Keywords:** Epigenetics, HPTMs, Cancer, Prognosis, Metastasis, Solid tumours

## Abstract

Phenotypic plasticity is a key mechanism of metastatic progression and cancer therapy resistance. This hallmark of human malignancies is enabled by highly conserved epigenetic mechanisms that control gene expression. Functional alterations in DNA methylation and histone post-translational modifications have been extensively described as drivers of metastatic dissemination and therapy resistance. Pharmacological inhibitors of epigenetic enzymes can revert these alterations, thereby stopping cancer progression and counteracting the emergence of resistant clones. Despite promising pre-clinical evidence, the clinical implementation of epigenetic therapies in solid cancers has led to disappointing results. Several factors can explain these challenges, including the lack of rational combinations. Notably, response to epigenetic treatments can be heterogeneous and short-lived. A liquid biopsy technology that allows the measure of specific epigenetic alterations enables patient selection and therapy monitoring, leading to the development of precision epigenetic therapies. In this review, we discuss the state of the art of this emerging treatment modality, and we identify key challenges that need to be overcome to reach the full potential of this new therapeutic concept.

## Introduction

Cancers are characterised by extreme phenotypic plasticity, i.e. by the ability to undergo rapid and dynamic phenotypic changes [1]. This feature enables drug resistance and metastasis, which are the two main causes of cancer-related deaths [2]. In this review, we will focus on targetable epigenetic alterations that cause cancer phenotypic plasticity. We will propose a new therapeutic paradigm to optimise the efficacy of epigenetic therapies in oncology, thereby blocking metastatic progression and reversing drug resistance. Although this paradigm can be applied to all solid cancers, we will focus on the three most prevalent malignancies (breast, prostate and lung) and sarcomas (the only solid cancer for which epigenetic therapies are approved), as proof-of-concept.

The discipline of epigenetics studies the heritable changes in cell phenotypes that do not result from alterations of the DNA nucleotide sequence [3]. The main epigenetic alterations are DNA methylation, DNA hydroxymethylation and histone post translational modifications (HPTMs). Here, we will focus on HPTMs, but most of the principles we discuss can be generalised to other epigenetic mechanisms.

The mammalian genome is structurally organised in DNA–protein complexes known as chromatin [4]. Chromatin exists in three main transcriptional states: [I] euchromatin, which is accessible to RNA polymerases and transcription factors and can therefore be actively transcribed; [II] constitutive heterochromatin, which is always inaccessible to transcription (e.g. centromeres and telomeres); [III] facultative heterochromatin, which is temporarily inaccessible to transcription. During development, lineage specification is underpinned by the dynamic regulation of euchromatic and facultative heterochromatic regions, which enable cell-specific gene expression profiles.

Nucleosomes are the fundamental units of chromatin and, therefore, play a key role in epigenetic gene regulation. Nucleosomes consist of an octamer of four main core histone proteins [H2A, H2B, H3 and H4] around which 147 bp of DNA are wrapped. Each adjacent nucleosome is separated by approximately 20–50 bp of free DNA. Nucleosome proteins are stabilised by histone H1. Each of the core histone proteins has a long N-terminal tail, which plays a key role in epigenetic gene regulation. These tails undergo numerous HPTMs including acetylation, methylation, phosphorylation and ubiquitination. The function of these HPTMs depends on the type of modification and the affected residue (Table 1). For example, histone acetylation is associated with an open chromatin status and therefore with gene transcription. Histone methylation can have opposite effects, depending on the affected residue. Lysine 27 of histone 3 (H3K27) can be mono-, di-, and tri-methylated (H3K27me, H3K27me2 and H3K27me3, respectively). This leads to gene silencing. On the other hand, histone H3K4me3 marks active transcription [5]. The combinatorial complexity of HPTMs generates more than 50 different modifications at different residues. It is important to note that HPTMs are not only involved in epigenetic gene regulation but also in DNA replication, repair, chromosome condensation and splicing [6]. The complex interaction between epigenetic and non-epigenetic functions of HPTMs is not fully elucidated.

**Table 1:** Examples of histone post-translational modification (HPTMs) with known function in transcription

Histone	HPTM site	Role in transcription	*References*
H3	K9ac	Activation	[7]
	K18ac	Activation	[8]
	K4me	Activation	[9]
	K4me2	Activation	[9]
	K4me3	Activation	[9]
	K9me	Repression	[9]
	K27me3	Repression	[10]
	K36me3	Activation	[11]
H4	K16ac	Activation	[12]
	K20me3	Repression	[12]

Some of the listed modifications control other functions, such as DNA repair and transcriptional elongation

**Fig. 1:**
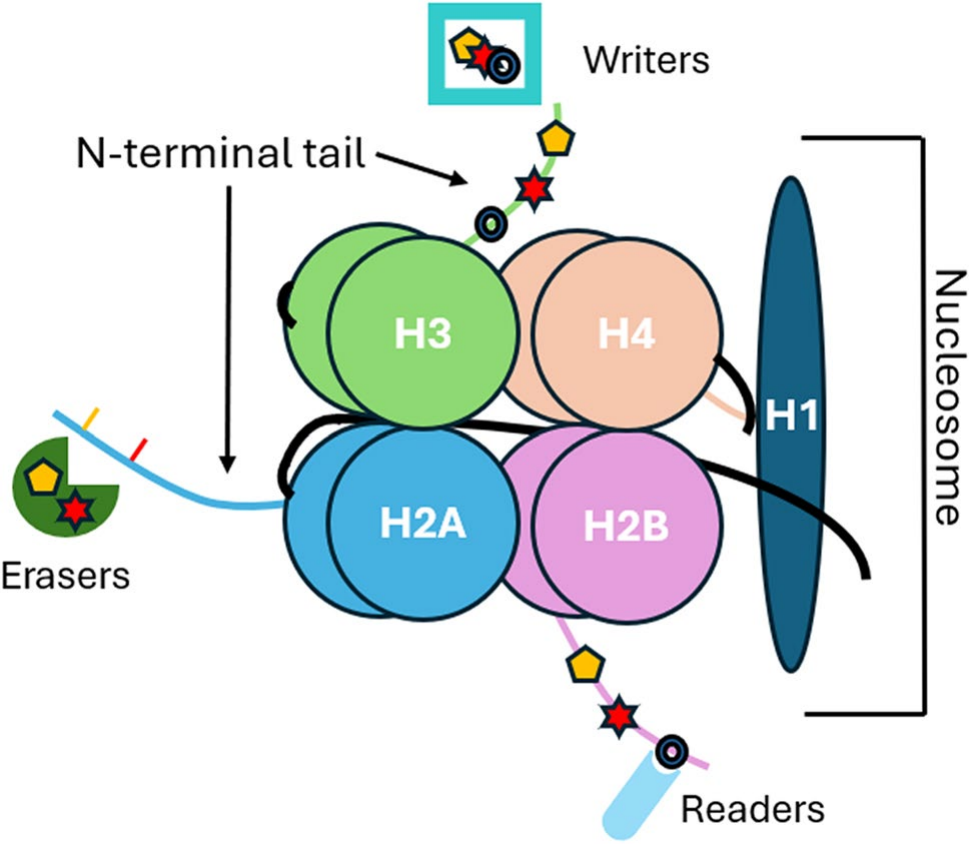
Schematic representation of the nucleosome, which is composed of an octamer of core histones (H2A, H2B, H3 and H4), which are stabilised by Histone H1 and 145–147 bp of DNA. Multiple different enzymes have a role in the addition and removal of histone modifications, such as acetylation (yellow hexagon) and methylation (red star). Writers and erasers can modify histones by allowing for the addition or removal of histone tail modifications, and readers can translate these modifications, which allows for distinct cellular profiles [16–18]

Each HPTM is maintained by the dynamic interaction of “writers”, “erasers” and ‘’readers’’ [13]. For example, the levels of H3K27me3 are determined by the opposite functions of EZH2 (histone methyltransferase-writer) and KDM6 (histone lysine demethylase-eraser) [14]. The EED “reader” binds to H3K27me3 and facilitates the activity of other epigenetic factors, thereby controlling gene expression [15]. The function of “writers”, “readers” and “erasers” is summarised in (Fig. 1).

During the human lifespan, epigenetic markers determine spatio-temporal differences in gene expression that underpin embryonic development, tissue specification and organ functions. For example, DNA methylation and HPTMs maintain chromatin stability, enable genetic imprinting and X-chromosome inactivation [19]. Furthermore, epigenetic alterations cause heritable variation of somatic cells’ gene expression patterns, resulting in distinct functions of diverse cell types. Notably, genome-wide patterns of DNA methylation and histone modifications form throughout early development and persist over numerous cell divisions [20].

## HPTMs in cancer drug resistance and metastasis

In addition to their physiological roles, HPTMs can be hijacked by cancer cells to promote oncogenesis. Numerous studies have shown that HPTMs play a key role in regulating both drug resistance and metastasis. Several mechanistical studies show that the metastatic progression and drug resistance share key molecular mechanisms of phenotypic plasticity. For example, epithelial to mesenchymal transition (EMT), an early step of the metastatic cascade, directly modulates the expression of apoptotic genes, thereby affecting drug resistance [21]. In addition, metastasis-initiating cells within the primary tumour have several features that enable drug resistance, including the ability to enter and exit dormancy, metabolic reprogramming and apoptosis resistance [22]. This body of evidence shows that metastasis and drug resistance are deeply interconnected phenomena, which are probably enabled by shared epigenetic mechanisms. For example, the H3K27me3 writer EZH2 has been identified among the most upregulated genes during the progression from localised to metastatic to hormone therapy-resistant prostate cancer [23]. In keeping with our hypothesis, EZH2 inhibition restores enzalutamide sensitivity in metastatic prostate cancer [PCa] [24]. Mechanistically, EZH2 modulates the expression of cell cycle and cell differentiation markers [25]. Some of these epigenetic effects are mediated by H3K27me3-dependent gene silencing. However, EZH2 also acts via histone methylase-independent mechanisms. For example, EZH2 methylates the ERG transcription factor, thereby facilitating its activation. This favours PCa cells’ invasiveness and promotes metastasis [26]. EZH2 has also been shown to promote epithelial-to-mesenchymal transition [EMT] and drug resistance in lung cancer [LC] [27]. EZH2 is also highly expressed in different sarcoma subtypes [28], and its silencing stops sarcoma cell proliferation [29]. Whilst EZH2 favours oncogenic progression in PCa, LC and sarcomas, its function in breast malignancies seems to be quite different. For example, Mieczkowska et al. [30] found that drug resistant breast cancer [BC] cells downregulate the expression of EZH2. This leads to the reactivation of genes that promote cancer cell survival. On the other hand, hypoxic conditions activate the XBP1 transcription factor in BC cells [31]. Hypoxia-activated XBP1 recruits EZH2 to the promoter of tumour suppressor genes, thereby favouring BC metastasis [32]. These examples highlight the key dependence of epigenetic marks on cellular and micro-environmental contexts. The involvement of HPTMs in metastasis and drug resistance is not limited to H3K27me3. For example, histone deacetylases (HDACs) are “erasers” that remove acetyl Marks, thereby silencing gene expression. The human genome encodes 18 different HDACs [33]. Each HDAC isoform displays slightly different substrate specificity and sub-cellular localization. Specific HDACs have been widely implicated in drug resistance and metastasis, mainly (but not exclusively) as oncogenes. For example, HDAC2 cooperates with EZH2 to induce the hypoxia-dependent metastatic response discussed above [31]. HDAC2 also promotes migration and invasion in LC [33]. The emerging role of other HDACs in oncogenic progression has been recently reviewed [34].

Taken together, this evidence suggests that HPTMs are key regulators of cancer metastasis and drug resistance but also that their effects are highly tissue- and microenvironment-dependent. Hence, epigenetic enzymes, such as erasers and writers are attractive therapeutic targets, but their clinical application needs a molecularly informed, tailored approach.

## Epigenetic therapies in solid cancers: pre-clinical evidence

**Fig. 2:**
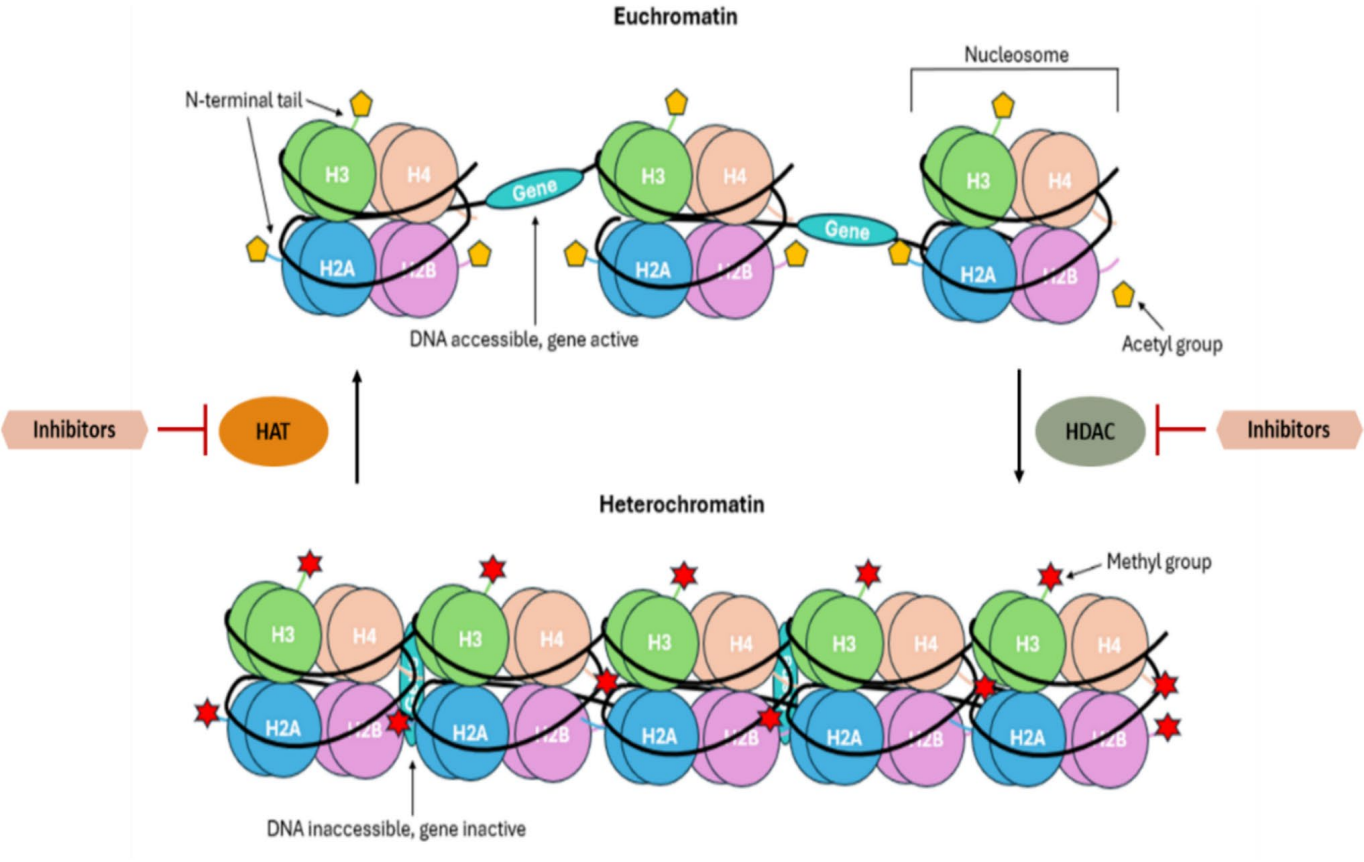
Histone deacetylases (HDACs) facilitate the removal of acetyl groups from lysine residues, resulting in condensation of chromatin (heterochromatin) and therefore repressing gene transcription by preventing access of transcriptional machinery to the DNA. HDAC inhibitors (HDIs) induce acetylation, which results in an open chromatin state and increasing gene expression. On the other hand, histone acetyltransferases (HATs) relax the chromatin structure, allowing for active gene transcription (euchromatin) [37–39]

HPTMs have been investigated as therapeutic targets for a broad range of diseases from solid tumours to inflammatory diseases. The role of HPTMs in the regulation of gene expression makes them a promising target for therapeutic intervention, and a few clinical trials are investigating the use of histone acetylation and histone methylation-targeted therapies [34]. As previously mentioned, histone acetyltransferases (HATs) and HDACs are involved in the acetylation of lysine residues [35], and histone methyltransferases (HMTs) and histone demethylases (KDMs) are involved in methylation of histone tails [35]. HDAC enzymes specifically regulate chromatin remodelling (Fig. 2) and are divided in four groups: Class I which are commonly located in the nucleus, Class II which shuttle between the nucleus and the cytoplasm, Class III which are mainly localised to the mitochondria but can be found within the nucleus and the cytoplasm and Class IV, which are present in the cell nucleus [36]. Both HDAC and HAT pharmacological inhibitors (HDACi and HATi) have been developed, but the former are more extensively used as anti-cancer drugs and have been approved for use in hematological cancers such as multiple myeloma and relapsed or refractory peripheral T-cell lymphoma (PTCL). Since most clinical studies in BC and LC assess the efficacy of HDACi, we will explore the pre-clinical activity of these compounds in the following two sections. The last two sections on PCa and sarcomas will also explore the role of some histone methyltransferase inhibitors (HMTi), since this class is more actively tested in clinical trials for this malignancy.

### HDAC inhibition in breast malignancies

HDAC enzymes have a role in transcriptional regulation of both the oestrogen and progesterone receptor pathways. HDACis, such as entinostat, have been shown to promote anti-tumour activity by inducing cell cycle arrest and apoptosis in pre-clinical ER-positive and triple-negative BC models (TNBC) [40]. Entinostat is a class I HDAC inhibitor and promotes hyperacetylation and transcriptional activation of specific genes, which can inhibit cell proliferation and apoptosis [41]. As for other cancers, the mortality of patients diagnosed with BC is almost always due to the metastatic spread of this disease. In TNBC models, treatment with entinostat reduced the population of tumour-initiating cells (TICs) and resulted in reduced mammosphere-forming ability, as well as reduced tumour formation *in vivo*. Importantly, dissemination of cancer cells was reduced following entinostat treatment, which also resulted in reduced metastatic colonization of the lung [42]. Metastasis is often enabled by EMT, which is characteristic of TNBC. *In vitro*, Shah and colleagues demonstrated that entinostat treatment can reverse EMT, which may reduce the formation of metastasis [43], and in patient-derived xenografts (PDXs), entinostat in combination with letrozole or exemestane showed greater anti-tumour activity, significantly reducing tumour growth rate, compared to treatment with the single agent [44]. Based on these promising pre-clinical results, several clinical trials explored the efficacy of entinostat in BC, as further discussed in Section 4.

Vorinostat (suberoylanilide hydroxamic acid — SAHA) is an oral HDACi of Classes I and II HDACs that was the first FDA-approved HDACi [44] and inhibits the catalytic activation of HDACs by binding to the zinc ion located in the enzyme catalytic domain [45] and promotes anti-tumour activity by inducing autophagy, cell cycle arrest, inhibition of proliferation and tumour growth reduction in both luminal and TNBC BC models [45]. Interestingly, it has been reported that vorinostat regulates receptors that are normally not expressed in TNBC, resulting in the re-expression of oestrogen receptor alpha (ERα) and progesterone receptor (PR) which inhibit both the growth of TNBC cells, and their sensitization to tamoxifen [46]. However, in MCF-7 cells, which are ERα and PR positive, vorinostat can deplete ERα at both transcriptional and post-translational for protein levels by inhibiting ERα gene expression and stimulating ubiquitin–proteasome pathway degradation, which could serve as a novel therapy for hormone-refractory BC [45].

It has been suggested that HDACis can exert potent anti-metastatic activity, but a handful of studies have indicated that HDACis can promote metastasis in other cancers. In a study by Hu et al. (2023), *in vivo* monotherapy with vorinostat resulted in morphological changes to BC cells, increased invasiveness and promoted LC metastasis. These results highlight the requirement for investigations into potential combination therapies to improve clinical efficacy of HDACis in BC, especially as promising pre-clinical results of monotherapy with HDACis, such as vorinostat, have not translated well to clinical studies, yielding negative results. However, pre-clinical and clinical studies investigating the efficacy of vorinostat in combination with anti-tumour drugs have shown promising results. For example, Mitchell et al. demonstrated synergistic lethality in BC cells when combining vorinostat with the cyclin-dependent kinase (CDK) inhibitor flavopiridol, and Min et al. identified increased sensitivity of olaparib in TNBC cells by inducing DNA damage and apoptosis.

### HDAC inhibition in lung malignancies

Similarly to BC, pre-clinical vorinostat monotherapy and combination therapy have been investigated extensively in both small cell lung cancer (SCLC) and non-small cell lung cancer (NSCLC). Pre-clinical monotherapy with vorinostat in NSCLC had a modest effect on anti-tumour activity [47]; however, the combination of vorinostat with chemotherapy (cisplatin) resulted in a synergistic augmented anti-tumour activity both *in vitro* and *in vivo* [47]. A study by Pan et al. investigated the efficacy of vorinostat and cisplatin in H209 and H146 SCLC cell lines, and as expected, cell growth inhibition was higher with combination treatment, compared to the single agents, suggesting the use of combination therapies can improve the chemotherapeutic outcomes of cisplatin [48]. This study also showed that vorinostat regulates the expression of some proteins that have crucial roles in enhancing combination therapy efficacy, including thymidylate synthase (TS), and histone H3 acetylation. TS is an essential enzyme for DNA synthesis in cancer cells, and upon inhibition, imbalances of deoxyribonucleotides arise and lead to DNA damage. Pan et al. observed that vorinostat downregulated protein levels of TS, and this inhibition remained upon combination with cisplatin, thereby suppressing cell growth. Additionally, acetyl histone H3 was significantly increased when combining vorinostat with cisplatin, allowing for more accessibility of transcriptional machinery to the DNA [48]. Additional combination therapies with vorinostat are being investigated in clinical trials, which are further discussed in Section 4.

Romidepsin (formerly FK228) is a depsipeptide, which is a natural cyclic peptide HDACi, which induces the expression of genes that are involved in cell growth inhibition, promotion of apoptosis and inhibition of both cell proliferation and angiogenesis [49]. *In vitro* studies have shown that romidepsin depletes expression of epidermal growth factor receptor (*EGFR*), erbB2 and Raf-1 kinases in NSCLC cells and resulted in time-dependent apoptosis in cells expressing wild-type (wt) or mutant *EGFR* [50]. Zhang and colleagues investigated the efficacy of romidepsin in combination with erlotinib, which is an epidermal growth factor receptor (*EGFR*) tyrosine kinase receptor (TKI) in nine NSCLC cell lines with varying *EGFR* and *KRAS* mutations. NSCLCs which contain *EGFR* mutations are often sensitive to TKIs, such as erlotinib, but some patients can develop resistance to these methods of treatment over time, and most wild-type *EGFR* NSCLCs are resistant to TKIs. Interestingly, combining romidepsin with erlotinib reduced the *IC*_50_ of erlotinib to < 2.5 µM in all cell lines, which was determined to be synergistic *in vitro*, enhancing apoptosis and inhibiting xenograft growth *in vivo*.

As reviewed elsewhere [51], immune checkpoint inhibitors (ICIs) are very promising immune-modulating drugs that target the PD-1 and/or PD-L1 proteins thereby boosting the anti-cancer immune response. Interestingly, HDAC and other epigenetic therapies seem to enhance the activity of immunotherapies through several mechanisms. For example, etinostat increases the efficacy of ICIs in a murine model of LC [52]. This effect is mediated by an epigenetic treatment-induced increase in IFN-γ-producing CD8^+^ T-cells with a decrease in intratumoral Treg cells. This result corroborates previous findings indicating synergism between other HDAC is and ICIs [53].

### Histone methylation modulators in prostate malignancies

**Fig. 3:**
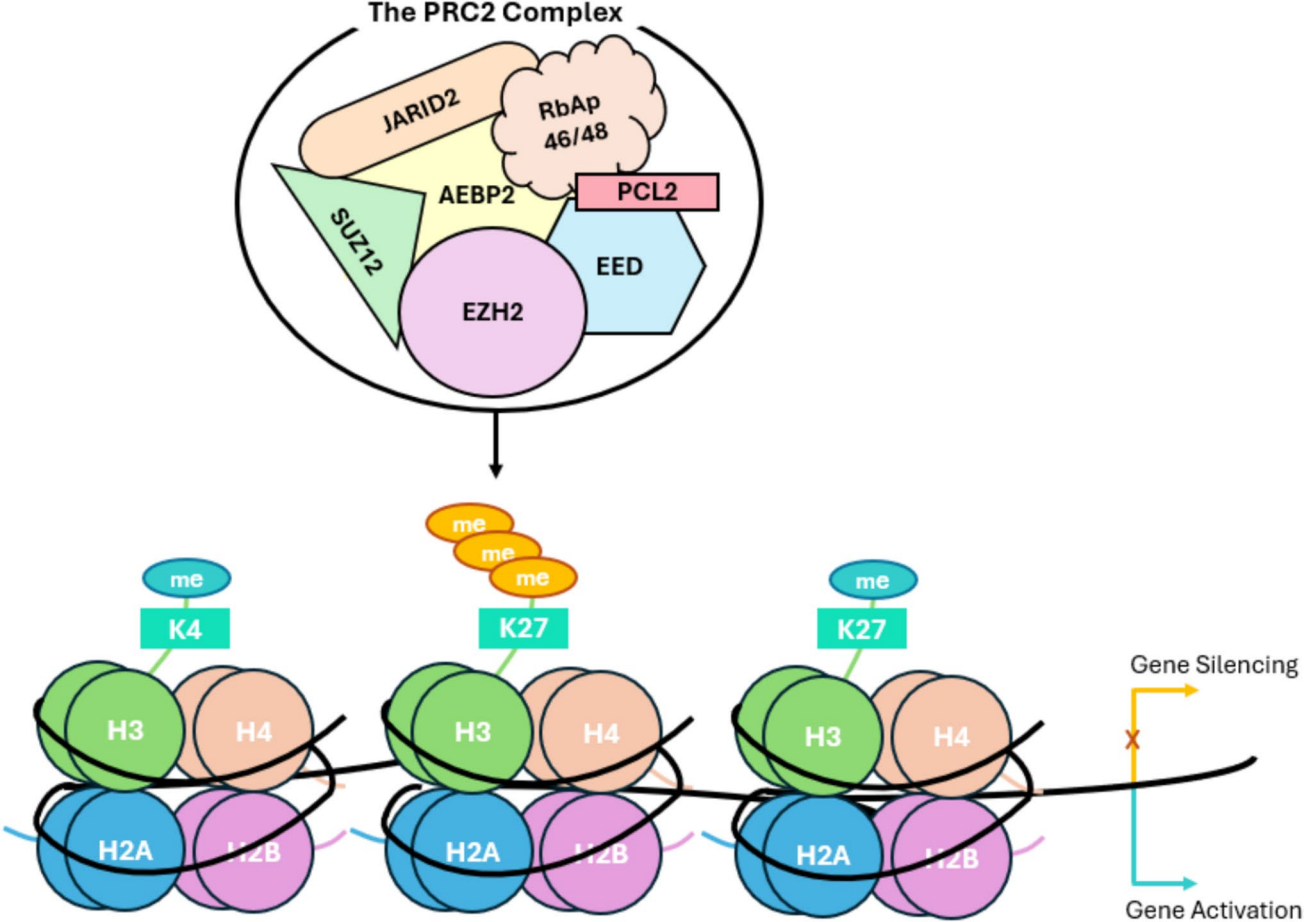
The polycomb repressive complex (PRC2) is responsible for the tri-methylation of Histone 3, at Lysine 27 (H3K27), which is associated with gene repression. The PRC2 complex has three Main subunits; the enhancer of zeste 2 (EZH2) which catalyses the addition of methyl groups to H3K27, the embryonic ectoderm (EED) which enhances this catalysis by recognising and binding to H3K27 and stabilising the PRC2 complex, and the suppressor of zeste 2 (SUZ12), which regulates the level of H3K27me3. EZH2 overexpression has been linked to the repression of tumour suppressor genes, and derepression of these genes has been identified in advanced cancers and metastasis. Therefore, components of the PRC2 complex have become popular targets for drug discovery, as abrogating H3K27me3 levels may allow for an inhibition of cell proliferation in cancer [64–68]

HPTM enzyme-targeting inhibitors have been extensively investigated in PCa. Due to its extensive pathogenic function, the histone methyltransferase EZH2 is an obvious therapeutic target, especially for advanced PCa, e.g. castration-resistant adenocarcinomas (CRPC) and malignancies with neuroendocrine features (NEPC) [54]. As shown in Fig. 3, EZH2 is a methyltransferase, which normally acts as part of the polycomb repressive complex 2 (PRC2) to catalyse H3K27me3 [55]. In addition to its PRC2-dependent function, EZH2 has non-canonical functions that are relevant to PCa progression, e.g. the co-activation of the androgen receptor [56]. Several EZH2 inhibitors (EZH2i) are currently in clinical trials, and some of them have been approved for the treatment of metastatic sarcomas and diffuse large B cell lymphomas [57]. Despite the progress in other malignancies, the use of EZH2i as monotherapy in advanced PCa did not yield promising results, causing cancer cell death only at very high concentrations [58]. A more promising strategy involves the use of EZH2i to reprogramme PCa cells and make them more susceptible to other therapies. For example, it has been shown that EZH2i restore enzalutamide sensitivity in advanced PCa cells [24]. This effect is potentiated by the ability of EZH2i to reactivate an anti-cancer immune response *in vivo* [59]. Similarly, HDACi showed limited potency as monotherapy in PCa models, but these compounds were often able to reverse drug-resistant phenotypes, e.g. by regulating the DNA repair pathways [60, 61]. Notably, the combination of EZH2 and HDACi seems to be highly synergistic in PCa pre-clinical models, by reactivating the expression of cellular stress genes [62]. An alternative drug target within the PRC2 complex is the EED reader, which has both polycomb-dependent and -independent functions [62]. Interestingly, small molecule EED inhibitor showed synergistic activity with androgen receptor pathway inhibitors (ARPI) in PCa pre-clinical models [63].

Another interesting eraser inhibitor is represented by histone demethylases, such as LSD1, which are also upregulated in PCa and promote PCa progression [69]. This suggests that both histone methylation (EZH2) and demethylation could be interesting therapeutic strategies in PCa. For example, a broad histone demethylase inhibitor was shown to be effective in inducing apoptosis in both androgen-responsive and androgen-insensitive PCa cells [70]. This effect was accompanied by broad metabolic reprogramming and seemed to be cancer selective.

Taken together, these pre-clinical results in BC, LC and PCa highlight the importance of selecting the right inhibitor for the right tumour subtype and the greater efficacy of rational combinations, especially in drug-resistant cancers.

Finally, PRMT5 was investigated as a new therapeutic target in PCa and other malignancies. This gene encodes a protein arginine N-methyltransferase [71], which is responsible for the mono- and symmetric dimethylation of arginine residues on histones H2A, H3 and H4 [72] by transferring methyl groups from S-adenosylmethionine (SAM) to a guanidine nitrogen of arginine resulting in S-adenosylhomocysteine (SAH) and methylarginine [72]. The methylation of histone arginine residues can result in gene activation or repression. For example, the dimethylation of H3 arginine 8 (H3R8) is associated with gene repression, and the dimethylation of H3R17 is associated with regulatory regions of active genes [73]. PRMT5 and its accompanying methyltransferase activity has been implicated in cancer tumorigenesis and as a marker of poor prognosis, suggesting an oncogenic role in many human cancers and making it an attractive therapeutic target for drug development [77;79]. Furthermore, it has been suggested that PRMT5 can maintain expression of genes by antagonizing PRC2-mediated transcriptional repression, which may allow for the development of combination treatments for oncology, which would include a PRMT5 inhibitor (PMRT5i) and a PRC2 inhibitor (PRC2i) [73]. The role of PRMT5’s function in PCa is unclear, and current research shows inconsistent findings. Deng et al. found that PRMT5 is an epigenetic activator of the AR in PCa cells, with knockdown of PRMT5 in AR + LNCaP suppressing the growth of xenograft tumours in mice, and inhibiting AR transcription [74]. However, a study by He et al. looked at PRMT5i in PCa, focusing on CRPC [75]. Here, inhibition of PRMT5 using EPZ015666 has a more prominent anti-cancer effect in PCa samples with increasing aggressiveness. For example, in AR+ LNCaP cells, proliferation is marginally affected, but AR PC3 cells are sensitive to PMRT5i. Interestingly, PRMT5i seems to induce differentiation, and reprogrammes PC3 from a basal to luminal state [75]. Finally, it is suggested that PRMT5i de-represses expression of immune genes in AR + and AR − PCa cells, which provides rationale for combined PRMT5i and immunotherapy, especially in inhibiting CRPC [75]. Furthermore, research suggests that PRMT5 is upregulated after radiation therapy, which is the standard treatment for locally advanced PCa, and epigenetically activates DNA damage repair (DDR] genes in response to double-stranded breaks in the DNA (DSB] caused by the ionizing radiation (IR). In 20–30% of patients who have received radiation therapy, cancer cells can acquire radio resistance, which is conferred by the activation of DDR pathways [76]. This can then allow for neuroendocrine differentiation, which can lead to the development of NEPC, an incurable malignancy [76]. JNJ-64619178, another promising PRMT5i, can downregulate the expression of DSB repair proteins in PCa cell lines including LNCaP and AR-independent lines such as DU145. Furthermore, JNJ-64619178 has been shown to inhibit the transdifferentiation of prostate adenocarcinoma by targeting IR-induced neuroendocrine differentiation and therefore shows promising potential as a radio sensitizing agent [76]. Currently, no clinical trials are investigating the use of PRMT5i and PCa (clinicaltrials.gov). However, there are five inhibitors (GSK3326595 [sister inhibitor of EPZ015666], JNJ-64619178, PF06939999, PRT543 and PRT811) in phase 1 clinical trials for other malignancies.

### Case study: EZH2 inhibitors in sarcomas

Due to their clinical success in sarcomas, we will focus this section on EZH2i. More than 10 years ago, pharmacological inhibition of EZH2 was identified as a viable therapeutic strategy for embryonal rhabdomyosarcomas [77]. Subsequent studies confirmed that EZH2i were also effective in osteosarcomas [78], Ewing sarcomas [79] and other subtypes. However, single-agent treatment with EZH2i mostly caused sarcoma differentiation and arrested cancer cell proliferation. Combining EZH2i with retinoic acid (a differentiating agent) induced stronger apoptotic response and more durable therapeutic effects [80]. These results confirm the need for rational combinations between PRC2 inhibitors and other targeted therapies, as discussed for PCa. This paradigm was reinforced by the discovery of a potent synthetic lethality interaction. It is well known that cancer cells with inactivating mutations in the SWI/SNF (switch sucrose non-fermentable) chromatin remodelling complex become addicted to PRC2 function [81]. The SWI/SNF complex consists of 15 subunits encoded by 29 genes. Some of these genes are onco-suppressive and are inactivated (e.g. via acquired gene deletion) in several sarcoma sub-types [82]. It is, therefore, conceivable that sarcomas with mutations in SWI/SNF genes could be more susceptible to EZH2i. This hypothesis was confirmed by a pre-clinical study that tested the efficacy of tazemetostat in sarcomas with genetically inactivated SWI/SNF complex [83]. In these conditions, EZH2 inhibition eradicates incurable sarcomas *in vivo*. As we will discuss in the clinical section, this result led to a successful clinical trial. Subsequent studies showed that resistance to EZH2i can occur even in SWI/SNF-mutated sarcomas. For example, a mutational analysis on SWI/SNF inactivated sarcomas exposed to tazemetostat, identified mutations in the *RB* (retinoblastoma) pathway as a key mechanism of resistance [84]. These acquired mutations overcome the cell cycle arrest induced by EZH2 inhibition. Aurora kinase B (AURKB) targeting bypasses the effect of *RB* mutations, thereby restoring the anti-cancer effect of tazemetostat. Hence, combining tazemetostat and AURKB-targeting compounds could be an effective strategy to prevent resistance to EZH2i in sarcoma and other malignancies.

## Epigenetic therapies in solid cancers: clinical evidence

As outlined in the previous paragraph, the encouraging pre-clinical results of HPTM enzyme-targeting agents in combination with cytostatic or cytotoxic agents constituted the rationale for clinical trials in different oncological populations. The first registered trials are dated in the late nineties: they were designed as early phases (i.e. phase I or I/II) and recruited patients with different types of solid malignancies, using first-generation HDAC is, such as valproic acid. This strategy provided the first clinical evidence on the activity and efficacy of epigenetic drugs in oncology [71]. In the last 15 years, epigenetic drugs have raised renewed interest, thanks to their combination with target therapies, such as ICIs and anti-human epidermal growth factor receptor 2 (HER2) agents. In the following paragraph, we will discuss the most relevant or advanced trials (i.e. phase II or III) of HPTM-targeting agents in the setting of BC, LC and PCa. The results from the positive trials cited in the following paragraphs are summarised in (Table 2).

**Table 2:** Activity or efficacy results from positive clinical studies cited across Section 4

Condition	Study reference; phase	Experimental drug	Study’s arms	Primary endpoint
**BC**	[92}; III	Entinostat	Exemestane + entinostat/placebo	PFS assessed by the independent radiographic committee (IRC): PFS was significantly longer in exemestane/entinostat group (HR 0.76; 95% *CI*, 0.58 − 0.98, *p* = 0.046)
	[85]; II	Vorinostat	Vorinostat + tamoxifen	*ORR* 19% (H0: 16.3%]
	[86]; I–II	Vorinostat	Paclitaxel ± trastuzumab (according to HER-2 status) + vorinostat	pCR rate HER2 +/TNBC: 54% (H0 30%) HR + : 0% (H0 15%)
**NSCLC**	[87]; II	Vorinostat	Carboplatin + paclitaxel + vorinostat/placebo	*ORR*: significantly higher for carboplatin + paclitaxel + vorinostat group (34%, 95% *CI*, 22%–47%, *p* = 0.02)
PC	[88]; Ib	Tazemetostat	Tazemetostat + abiraterone/prednisone or tazemetostat + enzalutamide	No dose-limiting toxicities

H0 is the predicted observation corresponding to the null hypothesis

*BC* breast cancer, *NSCLC* non-small cell lung cancer, *PC* prostate cancer, *PFS* progression-free survival, *CI* confidence interval, *HR* hazard ratio, *ORR* objective response rate, *pCR* pathological complete response

### Clinical investigation of HDAC inhibitors in breast malignancies

The most widely applied epigenetic drug class to BC is HDACi. Out of these, entinostat and vorinostat were tested in phase II or III clinical trials.

Entinostat was tested in hormone-positive (HR^+^), HER2 negative (HER2^−^ and HR^+^/HER2^−^) advanced BC patients who progressed after at least one line of endocrine agents. Two studies were conducted in the setting of Asiatic patients in Japan [89] and China [90], whilst a third study enrolled only Western patients [91]. Both the randomized Japanese phase II study and the Chinese phase III trial, compared exemestane plus entinostat vs. exemestane plus placebo. Both studies showed a clinically relevant improvement of median progression-free survival (mPFS) with the addition of the HDACi. The Japanese study did not reach global statistical significance. However, a subgroup analysis showed significant improvement in elderly women and patients selected based on circulating biomarkers. In particular, lower levels of histone lysine acetylation and higher inflammation Markers were associated with greater clinical benefit from the HDACi. The Chinese phase III study enrolled 354 HR^+^/HER2^−^ advanced BC patients in the same disease setting [90]. Entinostat improved mPFS in the HDAC-treated arm (Hazard ratio 0.76; 95% *CI*, 0.58–0.98; *p* = 0.046], with consistent benefit across all planned subgroups (e.g. age, visceral metastases and previous therapy regimens]. As expected, the safety profile of entinostat showed a higher rate of haematological toxicity, increased cholestasis and nausea. However, when the entinostat plus exemestane treatment was tested in Western HR^+^/HER2^−^ advanced BC population, efficacy results were not confirmed (mPFS placebo vs. entinostat: 3.1 vs. 3.3 months, *p* 0.30; median overall survival entinostat vs. placebo: 23.4 vs 21.7 months, *p* 0.94]. These results are puzzling because the drug’s target engagement was confirmed by a 1.5 increase in histone lysine acetylation in white blood cells upon entinostat treatment.

Similarly, the HDACi vorinostat has been evaluated in the setting of hormonal resistance and HER2 positivity. Trial development with this drug terminated at an earlier stage due to less promising results than entinostat. For example, the addition of vorinostat to tamoxifen, after evidence of disease progression upon endocrine therapy, showed an objective response rate (*ORR*) of 19% and a clinical benefit rate of 40% at 6 months [85]. Interestingly, this study used ^18^F-fluoroestradiol PET imaging to elucidate the potential mechanism of endocrine sensibility restoration by vorinostat. This investigation revealed no dynamic changes of ^18^F-fluoroestradiol during vorinostat administration [92], posing doubts on the scientific rationale of this strategy.

In the setting of trastuzumab-resistant HER2^+^ BC, the addition of vorinostat to trastuzumab demonstrated a low response rate, causing early study termination [93]. More promising results were seen in neoadjuvant chemotherapy of HER2^+^ locally advanced BC. In this context, vorinostat significantly increased the efficacy of standard chemotherapy (paclitaxel + trastuzumab followed by epirubicin plus cyclophosphamide) [86].

As suggested by pre-clinical evidence, HPTMs targeting agents need to be combined with other active agents in BC to demonstrate some activity. Monotherapies are instead generally ineffective [94, 95].

At present, epigenetic drugs have not found a place in the therapeutic armamentarium of BC which is rapidly evolving and progressively more enriched in biomarker-driven therapies. Nonetheless, epigenetic drugs are being studied in combination with newer backbone agents such as ICIs [96], providing a potentially new rationale for further investigation.

### Clinical investigation of HDAC inhibitors in lung malignancies

Most clinical trials involving HPTM-targeting drugs in the setting of NSCLC and SCLC were completed between 2005 and 2015 and stopped at the early stages of phase I/II, due to lack of activity [97, 98]. However, it is worth noting that some minimally active compounds showed promising results in terms of disease control. For example, vorinostat second Line monotherapy demonstrated a disease control rate of 57%, with mPFS and mOS of 2.3 and 7.1 months, respectively in NSCLC [97]. These results suggest that vorinostat should be tested in combination with other known active agents in LC.

A significant increase of anti-cancer activity was found with the addition of vorinostat over placebo to first line carboplatin/paclitaxel chemotherapy in 94 enrolled patients [87]. The increase in *ORR* from 12.5% (placebo) to 34% (entinostat) in the overall-treated population (*p* 0.02] was consistent across treated patients with different histologies (entinostat vs. placebo, respectively 33% vs. 0% for squamous LC, 34% vs. 16% for non-squamous LC) and associated with a non-significant increase in mPFS (entinostat vs. pleacebo 6.0 vs. 4.2 months, *p* 0.48] and mOS (entinostat vs. placebo: 13 vs. 9.7 months, *p* 0.17). However, a caveat for toxicities was raised, due to the more frequent grade 2–3 events in patients exposed to vorinostat, compared to placebo, and the recording of two toxic deaths that may be related to the experimental treatment.

Conflicting results were obtained with the addition of vorinostat to first generation epidermal growth factor receptor (EGFR) tyrosine kinase inhibitors (TKIs). The trials took place in the setting of pretreated NSCLC and were based on the biological rationale of EGFR sensitivity restoration with HDACi, in pre-clinical investigations. In the phase I/II study of vorinostat plus gefitinib in the EGFR-mutation unselected pretreated population [99], the HDACi combination strategy failed to meet the primary endpoint of 6.5 months mPFS, while it reached 9.1 months mPFS in the EGFR-positive subgroup. The latter benefit might be driven by the anti-EGFR drug. This hypothesis is confirmed by the observed lack of benefit of adding vorinostat to erlotinib in EGFR-positive erlotinib-refractory NSCLC patients [100].

More recently, the combination of HDACi with anti PD-1 pembrolizumab was tested, based on the pre-clinical rationale discussed earlier [101]. When vorinostat was added to pembrolizumab in the setting of pretreated NSCLC [101], no safety warning was raised, with low-grade nausea, vomiting, fatigue and haematological toxicities being the most frequent adverse events. This study included two populations of patients based on treatment with pembrolizumab: ICI-naïve and ICI-treated. In the ICI-naïve cohort, disease control rate was 83%, whilst in the ICI-treated, it was 58%. These results suggest that the activity of the combination is partially preserved, despite progression upon ICI treatment. This study also investigated potential pharmacodynamic biomarkers. Neither circulating myeloid-derived suppressor cells (MDSCs), nor tissue PDL-1 positivity correlate with treatment benefit. However, higher stromal CD8^+^ T cells seemed to predict better pembrolizumab/vorinostat activity.

The ENCORE 601 study [102], testing entinostat plus pembrolizumab in the setting of ICI-pretreated NSCLC, showed a lower-than-expected disease control rate.

The above-mentioned studies are not directly interpretable, considering the rapidly evolving therapeutic scenario of NSCLC. However, epigenetic drug-based combinations warrant exploration as the second line setting, which represents an unmet clinical need.

### Clinical investigation of histone methylation modulators in prostate malignancies

Treatment options for either hormone sensitive (HSmPC) or castration resistant (CRPC) advanced PCa have significantly expanded since 2000. Hence, most clinical trials tested HPTM-enzyme targeting drugs in combination with partners that no longer represent standard of care options. In contrast, more up-to-date combinations are still under evaluation, as discussed in this section.

Positive preliminary signals can be found in newer drug combinations comprising EZH2i. Of interest for further development is the combination between tazemetostat and either abiraterone acetate or enzalutamide in CRPC: in a phase Ib trial PSA-reduction was seen in 46% of patients receiving enzalutamide-based combination and in 14% in those receiving abiraterone, with a global disease control rate of 47% [88]. Part 2 A of a multi-cohort phase I trial testing the anti-EZH2 compound PF-06821497 [103], studied testing the anti-EZH2 compound PF-06821497 in combination with enzalutamide plus androgen deprivation therapy in CRPC patients. Among the 32 treated patients, *ORR* and mPFS were 14.3% and 9.7 months (95% *CI* 4.0–17.0), respectively, paving the way for the ongoing phase II trial in this disease setting [104]. Moreover, among the new scenarios in which anti-EZH2 combinations are being attested in CRPC, tazemetostat combination with PARP-inhibitors seems to leverage a strong biological rationale [105]. It is worth noting that some EZH2i that showed promising pre-clinical activity were discontinued upon disappointing early phase clinical trials. This was mainly due to poor pharmacokinetic profiles. For example, GSK-126 was tested in a cohort of patients with solid tumours or lymphomas. A phase I clinical trial failed to identify a dose that caused effective exposure to the drug, mainly because of a short half-life [106]. Interestingly, EZH2 activity can be downregulated also indirectly, through the inhibition of other polycomb-repressive complex 2 subunits. Of these, EED subunit inhibition represents a newer venue to tackle EZH2 pathogenic role in PC (NCT05413421).

As discussed in the pre-clinical section, the histone demethylase LSD1 is another promising target for PCa. Currently nine small molecule LSD1 inhibitors are being tested in early phase clinical trials for different conditions reviewed in [107]. One of these studies is recruiting PCa patients that failed treatment with enzalutamide. This proof-of-biology study will assess whether Pulrodemstat (an oral LSD1 inhibitor) can restore AR expression and therefore hormonal therapy activity in these patients. Other studies showed that Pulrodemstat has an acceptable safety profile: thrombocytopenia was the most common treatment‐related adverse event caused by this compound [108]. This adverse event was successfully managed with dose modifications.

Finally, we could not find any active clinical trial recruiting PCa patients for PMRT1 inhibitor treatment. However, some small molecule PMRT1 inhibitors are being tested in other malignancies and showing acceptable toxicity profiles [109]. Given the rationale discussed in the pre-clinical section, we believe that the combination of PMRT1 inhibitors and immunotherapy could be promising in PCa patients.

In conclusion, combination therapies with EZH2i seem to be the most promising option for advanced PCa. Given the strong biological rationale [69], we suggest that also LSD1 inhibitors should be explored in this setting.

### Case study: tazemetostat approval in sarcomas

Based on the compelling pre-clinical evidence discussed in Section 3.4, a phase II clinical trial investigated the efficacy of tazemetostat in advanced epithelioid sarcomas [110]. Although surgical resection of epithelioid sarcoma can be curative, this Malignancy shows a higher than 50% rate of local and metastatic recurrence [111]. Upon recurrence, epithelioid sarcoma is associated with very poor prognosis (mOS shorter than 2 years). Epithelioid sarcomas were selected for the high rate of inactivating mutations in the SWI/SNF gene, *SMARC1B* (40–90%] [112], which as discussed may predispose for increased EZH2i activity. Locally advanced and metastatic epithelioid sarcomas can be treated with palliative intent, using cytotoxic chemotherapy or TKIs [113]. When these therapies are administered as first line, reported *ORR*s range between 15 and 22%, whilst reported mPFS is between 2.5 and 6.7 months. When tazemetostat was administered as first-line therapy to patients with molecularly confirmed *SMARC1B* inactivation, *ORR* was 25%, and the mPFS was 9.7 months [110]. These results suggest clinical activity that May be higher than currently employed treatments. In some patients, response to tazemetostat was not observed until almost 4 months of treatment. This highlights the importance of prolonged treatments to reprogram the epigenome of solid tumours. Less encouraging results were obtained in patients that had been previously treated with other therapies. In these cases, tazemetostat induced a lower *ORR* (8%) and shorter PFS (3.4 months) which were comparable to the activity of other compounds. The toxicity profile of tazemetostat was favourable, suggesting that this epigenetic treatment could be coupled with other active agents. This evidence led to the FDA approval of tazemetostat for the treatment of advanced epithelioid sarcoma with *SMACA1B* inactivation [114].

Based on these encouraging results, several other studies were initiated in sarcoma and in other malignancies, to test the efficacy of tazemetostat in patients with SWI/SNF mutated tumours. Unfortunately, the results were not always positive. For example, a recent study on paediatric cancers (mostly sarcomas) with SWI/SNF mutations reported that tazemetostat caused a 5% *ORR*, which was statistically indistinguishable from the null hypothesis [115]. This negative result highlights the need to identify better strategies to select patients that may benefit from epigenetic treatments.

## Precision epigenetic therapies

Despite a strong biological rationale for the use of epigenetic therapies in oncology, so far HPTM-targeting compounds have had very limited clinical applications in solid tumours. The only exception to this pattern is tazemetostat in advanced epithelioid sarcomas [114]. As discussed, the discovery of this effective therapeutic option is underpinned by a genetically driven synthetic lethality mechanism. This success underlines the need for better patient selection, particularly through the development of non-invasive biomarker strategies like liquid biopsies.

### Circulating epigenetic markers and tumour biology

In the case of epithelioid sarcomas, patient selection is achieved by DNA analysis from tumour samples (e.g. biopsies), an invasive procedure that is not always feasible, especially for advanced cancer patients. A less invasive approach is provided by liquid biopsy biomarkers, i.e. cancer-derived molecules that are detected and measured in peripheral blood or other biological fluids. Traditionally, liquid biopsies focused on detection of cancer-specific genetic variations (e.g. copy number alterations, point mutations). However, genetic alterations are irreversible and not directly linked to the activity of epigenetic drugs. An epigenetics-focused liquid biopsy would enable the dynamic monitoring of cancer-derived biomarkers, which could be immediately correlated to the activity of epigenetic drugs. For this reason, several groups are testing methodologies for detection of cancer-derived epigenetic alterations from blood samples. The first technique of this type was a DNA methylation-based liquid biopsy, which proved to be effective in risk stratification of common malignancies, including BC, LC and PCa [116]. In these cancers, circulating DNA methylation patterns predict prognosis and correlate with relevant clinical parameters (e.g. grade or stage). Even if DNA methylation interacts with some HPTMs [117], the two mechanisms are not invariably linked, and changes in the former do not always reflect the status of the latter. Hence, DNA methylation-based liquid biopsies are not ideal tools to measure the activity and efficacy of HPTM-targeting drugs. Based on this evidence, volition developed a platform (Nu.Q®) that enables the simultaneous detection of up to 14 HPTMs from approximately 1 mL of plasma. We showed that cf-nucleosomes are more abundant in the plasma of cancer patients, compared to cancer-free individuals [118]. Our platform (Nu.Q®) enables the simultaneous detection of up to 14 HPTMs from approximately 1 mL of plasma. As proof of principle, we employed this technology in a curated cohort of hepatocellular carcinoma (HCC) patients [119]. Through bioinformatic analyses, we found that intra-tumoral expression of two HPTM enzymes was highly correlated with the survival of HCC patients exposed to the TKI sorafenib; high *EZH2* expression and low *SETD2* expression were both associated with worse prognosis. EZH2 catalyses the H3K27me3 repressive mark, whilst SETD2 catalyses the H3K36me3 activating mark. We therefore measured circulating levels of H3K27me3 and H3K36me3 in patients with HCC. Our results showed that total cf-nucleosomes were reduced from baseline to best response, confirming that most of the circulating nucleosomes are likely to derive from cancer cells shedding into the circulation. In line with our bioinformatic predictions, the ratio of circulating H3K27me3/H3K36me3 predicted patient survival (higher ratio = shorter survival). Pre-clinical studies showed that EZH2 inhibition increases the anti-cancer activity of sorafenib in HCC models [120]. Hence, it is conceivable that HCC patients with high levels of H3K27me3 could benefit from sorafenib-EZH2i combinations. These proof-of-principle results prompted us to test the Nu. Q technique in clinical cohorts exposed to HPTM-targeting drugs. For example, we are currently exploring the predictive efficacy of circulating H3K27me3 levels advanced in solid-cancer patients (i.e. pancreatic ductal adenocarcinoma, colorectal cancer and soft-tissue sarcomas) with the EZH2i tazemetostat in combination with immunotherapy [121].

The Nu. Q technique enables the detection of total circulating HPTM levels. Recently, some researchers have used circulating epigenetic biomarkers to predict the expression of specific genes in cancer cells. Chromatin immunoprecipitation (ChIP)-sequencing is a genome-wide technique that enables the detection of HPTMs at specific promoter sites. Traditionally, ChIP-Seq could only be performed on samples that generated large amounts of chromatin (e.g. cell lines). Baca et al. [122] used ChIP-seq to measure H3K4me3, H3K27ac and DNA methylation, in plasma from 433 individuals with one of 15 cancers. The combined use of activating and repressive epigenetic marks enabled them to accurately infer gene expression. In turn, this allowed the prediction of drug sensitivity and drug vulnerability. For example, the authors identified a subset of PCa patients with neuroendocrine features. These malignancies are resistant to commonly used hormonal therapies but could respond to platinum-based chemotherapy [123]. Notably, some of these highly resistant tumours expressed high levels of targetable epigenetic enzymes, such as EZH2.

The detection of elevated levels of specific circulating epigenetic markers, such as H3K27me3 or H3K36me3, raises critical questions: does this signify cancer dependency on the associated biological pathway, and if so, does it predict therapeutic sensitivity? Our data suggest that elevated circulating H3K27me3 levels might indicate overexpression of EZH2 in cancer cells, suggesting potential sensitivity to EZH2 inhibitors like tazemetostat. However, such correlations are not universally straightforward and should be confirmed in different clinical settings. The biological context and tumour microenvironment play critical roles. For example, elevated levels of an epigenetic marker in plasma might also result from tumour cell turnover or stress responses rather than direct correlation with intracellular cancer levels. Therefore, while these markers hold promise for stratifying patients, their interpretation requires integration with cancer-specific genomic, transcriptomic and proteomic data.

### Epigenetic reprogramming

The use of chemotherapeutic agents to treat cancer in the 1940 s identified an urgent need for combination therapies, as cancers were only temporarily entering remission. However, drug development is costly, time-consuming and labour-intensive [124]. The idea of combination therapies was first conceptualized in 1965 by Emil Frei, James F. Holland and Emil J. Freireich, and this involves the combination of two, or more, therapeutic agents which commonly have different mechanisms of action [125, 126]. These combinations should have a synergistic, or additive, effects allowing for a lower therapeutic dose, as they can enhance effectiveness of other anti-cancer drugs, reduce resistance and have relatively mild side effects due to dose reduction [124]. Epigenetic inhibitors are ideal candidates for combination therapies. As discussed in the previous sections, epigenetic inhibitors in combination with other therapeutic agents can reverse epigenetic dysregulation, overcome resistance and improve tolerance to traditional chemotherapeutic agents [127]. These effects are known as the epigenetic reprogramming of cancer cells. An excellent example of epigenetic reprogramming involves the rational use of epigenetic inhibitors and immunotherapies. Immunotherapy are a first-line treatment option for several cancer types [128], and this approach aims to enhance the natural anti-cancer immune response [129]. However, acquired or constitutive resistance to immunotherapies is a common occurrence in several cancers [130]. Using epigenetic inhibitors in combination with immunotherapies is a promising strategy for cancer treatment, as cancers can develop reversible epigenetic alterations (e.g. silencing of antigen-presenting genes) to evade the host immune system [131]. Hence, epigenetic inhibitors can be used to suppress the expression of genes involved in this immune evasion mechanism, allowing the tumour to be more vulnerable to immune attack [132, 133].

### Precision epigenetics pipeline

Taken together, this evidence suggests that we may be at the beginning of a precision epigenetics era. This paradigm shift is enabled by three main technical advances:

1. A pharmacopeia of safe and specific epigenetic drugs, which target specific HPTMs, as described in the previous sections
2. HPTM-targeted liquid biopsies
3. Pre-clinical data from synthetic lethality studies, which identify optimal combinations between HPTM inhibitors and other anti-cancer drugs, as detailed in paragraph 5.2

**Fig. 4:**
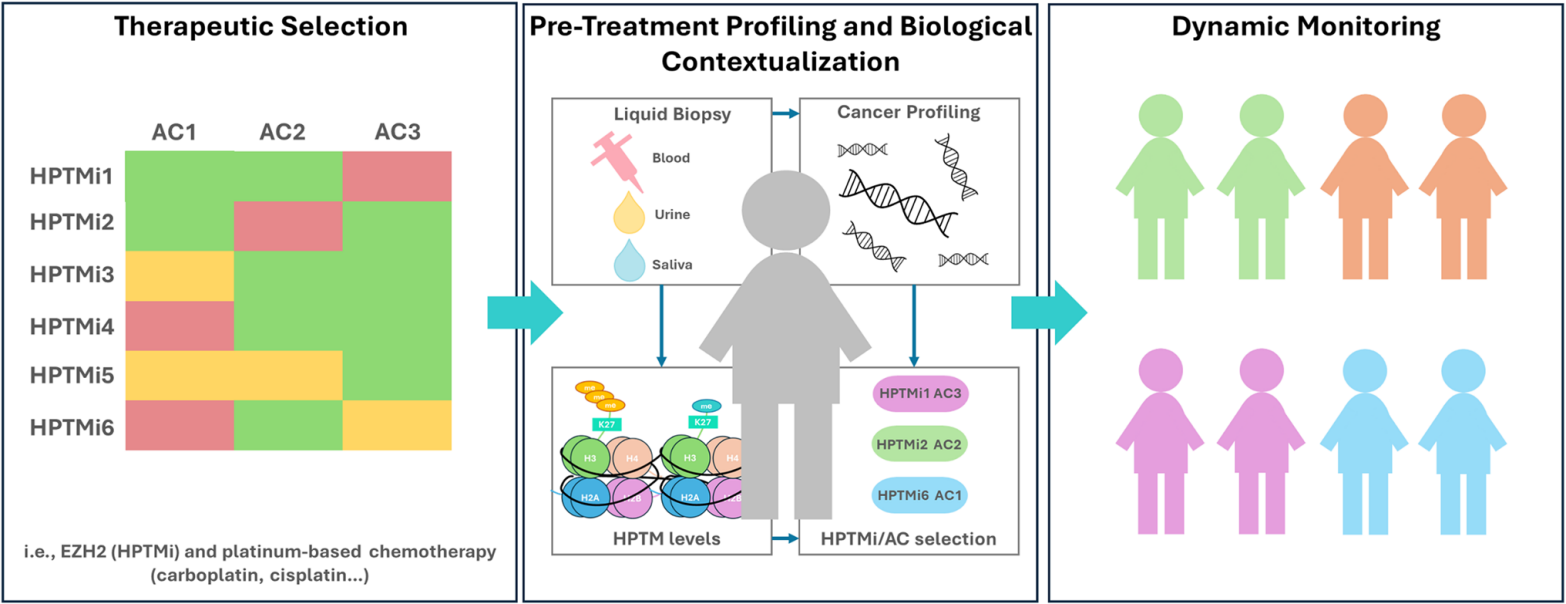
Precision epigenetics pipeline proposed for tailored patient selection for epigenetic therapies. Therapeutic selection should be used to identify HPTM inhibitors (HPTMi), such as tazemetostat, an EZH2i, and anti-cancer compounds (AC) that, when combined, have a synergistic or strongly additive relationship (red), such as HPTMi1 and AC3, compared to weakly additive (yellow), or antagonistic (green). Pre-treatment profiling and biological contextualisation should then be used to identify patients that have cancers with overexpression of HPTMS, using techniques such as ChIP-Seq or ATAC-Seq, which allows for the unique epigenome of each patient to be identified, and an appropriate combination treatment to be selected. At this stage, circulating levels of HPTMs should be correlated with intra-tumoral epigenetic activity. This will then lead to patient selection and dynamic monitoring (e.g. early identification of relapses via liquid biopsies

With this framework, we propose the precision epigenetics pipeline illustrated in Fig. 4. A precision approach combining liquid biopsy-derived epigenetic markers and pre-clinical data on synthetic lethality could significantly improve patient selection for HPTM-targeting therapies. This pipeline involves:

1. *Pre-treatment profiling*: Non-invasive measurement of circulating epigenetic markers (e.g., H3K27me3) to assess baseline tumour biology. Advanced techniques like cf-nucleosome profiling or ChIP-seq of plasma-derived chromatin fragments may enable fine-grained detection of relevant markers.
2. *Biological contextualization*: Correlation of circulating marker levels with tumour dependency on the corresponding epigenetic pathway. For example, high plasma H3K27me3 levels may warrant further analysis to confirm tumour EZH2 overexpression and PRC2 dependency.
3. *Therapeutic selection*: Patients with confirmed marker-tumour dependency are allocated to rationally designed combination therapies involving HPTM inhibitors (e.g. EZH2 inhibitors with ICIs or cytotoxic agents). These therapies should be selected by comprehensive pre-clinical drug screening.
4. *Dynamic monitoring*: Post-treatment evaluation of circulating marker changes to confirm target engagement and predict therapeutic outcomes. For instance, a reduction in circulating H3K27me3 upon EZH2 inhibitor treatment would suggest successful inhibition of the targeted pathway.

While promising, this approach faces technical and clinical challenges. These include ensuring the sensitivity and specificity of liquid biopsy techniques for low-abundance epigenetic modifications, standardizing methodologies and validating predictive markers in large, prospective clinical trials. Furthermore, it remains essential to establish direct causal links between circulating marker levels and tumor dependency to avoid misinterpretation.

In conclusion, we believe that by leveraging circulating epigenetic markers, we may be on the cusp of a precision epigenetics era. This paradigm shift may enable tailored therapies based on individual tumour biology, dynamic monitoring of treatment responses, thereby reducing unnecessary exposure to ineffective treatment.

## Data Availability

No datasets were generated or analysed during the current study.

## References

[1] Sheng, S., Margarida Bernardo, M., Dzinic, S. H., Chen, K., Heath, E. I., & Sakr, W. A. (2018). Tackling tumor heterogeneity and phenotypic plasticity in cancer precision medicine: Our experience and a literature review. *Cancer Metastasis Reviews,**37*(4), 655–663. 10.1007/s10555-018-9767-4 30484007 10.1007/s10555-018-9767-4 PMC6853189

[2] Kilmister, E. J., Koh, S. P., Weth, F. R., Gray, C., & Tan, S. T. (2022). Cancer metastasis and treatment resistance: Mechanistic insights and therapeutic targeting of cancer stem cells and the tumor microenvironment. *Biomedicines,**10*(11), Article 2988. 10.3390/biomedicines10112988 36428556 10.3390/biomedicines10112988 PMC9687343

[3] Dupont, C., Armant, D. R., & Brenner, C. A. (2009). Epigenetics: Definition, mechanisms and clinical perspective. *Seminars in Reproductive Medicine,**27*(5), 351–357. 10.1055/s-0029-1237423 19711245 10.1055/s-0029-1237423 PMC2791696

[4] Ueberheide, B. M., Mollah, S., & Garcia, B. A. (2024). On the hunt for the histone code. *Molecular and Cellular Proteomics,**23*(12), Article 100873. 10.1016/j.mcpro.2024.100873 39489218 10.1016/j.mcpro.2024.100873 PMC11696663

[5] Yang, L., Jin, M., & Jeong, K. W. (2021). Histone H3K4 methyltransferases as targets for drug-resistant cancers. *Biology,**10*(7), Article 581. 10.3390/biology10070581 34201935 10.3390/biology10070581 PMC8301125

[6] Xu, Y., Zhang, S., Lin, S., Guo, Y., Deng, W., Zhang, Y., & Xue, Y. (2017). WERAM: A database of writers, erasers and readers of histone acetylation and methylation in eukaryotes. *Nucleic Acids Research,**45*(D1), D264–D270. 10.1093/nar/gkw1011 27789692 10.1093/nar/gkw1011 PMC5210520

[7] Kutateladze, T. G. (2011). Snapshot: Histone readers. *Cell,**146*(5), 842-842.e1. 10.1016/j.cell.2011.08.022 21884941 10.1016/j.cell.2011.08.022 PMC3645985

[8] Hake, S. B., & Allis, C. D. (2006). Histone H3 variants and their potential role in indexing mammalian genomes: The “H3 barcode hypothesis.” *Proceedings of the National Academy of Sciences of the United States of America,**103*(17), 6428–6435. 10.1073/pnas.0600803103 16571659 10.1073/pnas.0600803103 PMC1564199

[9] Loyola, A., Bonaldi, T., Roche, D., Imhof, A., & Almouzni, G. (2006). PTMs on H3 variants before chromatin assembly potentiate their final epigenetic state. *Molecular Cell,**24*(2), 309–316. 10.1016/j.molcel.2006.08.019 17052464 10.1016/j.molcel.2006.08.019

[10] Yu, J., Yu, J., Rhodes, D. R., Tomlins, S. A., Cao, X., Chen, G., Mehra, R., Wang, X., Ghosh, D., Shah, R. B., Varambally, S., Pienta, K. J., & Chinnaiyan, A. M. (2007). A polycomb repression signature in metastatic prostate cancer predicts cancer outcome. *Cancer Research,**67*(22), 10657–10663. 10.1158/0008-5472.CAN-07-2498 18006806 10.1158/0008-5472.CAN-07-2498

[11] Xiao, C., Fan, T., Tian, H., Zheng, Y., Zhou, Z., Li, S., Li, C., & He, J. (2021). H3K36 trimethylation-mediated biological functions in cancer. *Clinical Epigenetics,**13*(1), 199. 10.1186/s13148-021-01187-2 34715919 10.1186/s13148-021-01187-2 PMC8555273

[12] Fraga, M. F., Ballestar, E., Villar-Garea, A., Boix-Chornet, M., Espada, J., Schotta, G., Bonaldi, T., Haydon, C., Ropero, S., Petrie, K., Iyer, N. G., Pérez-Rosado, A., Calvo, E., Lopez, J. A., Cano, A., Calasanz, M. J., Colomer, D., Piris, M. A., Ahn, N., … Esteller, M. (2005). Loss of acetylation at Lys16 and trimethylation at Lys20 of histone H4 is a common hallmark of human cancer. *Nature Genetics,**37*(4), 391–400. 10.1038/ng1531 15765097 10.1038/ng1531

[13] Allis, C. D., & Jenuwein, T. (2016). The molecular hallmarks of epigenetic control. *Nature reviews. Genetics,**17*(8), 487–500. 10.1038/nrg.2016.59 27346641 10.1038/nrg.2016.59

[14] Hemming, S., Cakouros, D., Isenmann, S., Cooper, L., Menicanin, D., Zannettino, A., & Gronthos, S. (2014). EZH2 and KDM6A act as an epigenetic switch to regulate mesenchymal stem cell lineage specification. *Stem cells [Dayton, Ohio],**32*(3), 802–815. 10.1002/stem.1573 24123378 10.1002/stem.1573

[15] Zhang, M., Li, J., Wang, Q., Urabe, G., Tang, R., Huang, Y., Mosquera, J. V., Kent, K. C., Wang, B., Miller, C. L., & Guo, L. W. (2023). Gene-repressing epigenetic reader EED unexpectedly enhances cyclinD1 gene activation. *Molecular Therapy-Nucleic Acids,**31*, 717–729. 10.1016/j.omtn.2023.02.024 36923952 10.1016/j.omtn.2023.02.024 PMC10009644

[16] Morales, V., & Richard-Foy, H. (2000). Role of histone N-terminal tails and their acetylation in nucleosome dynamics. *Molecular and Cellular Biology,**20*(19), 7230–7237. 10.1128/MCB.20.19.7230-7237.2000 10982840 10.1128/mcb.20.19.7230-7237.2000 PMC86277

[17] Yang, T., Liu, Z., & Li, X. D. (2015). Developing diazirine-based chemical probes to identify histone modification ‘readers’ and ‘erasers.’ *Chemical Science,**6*(2), 1011–1017. 10.1039/c4sc02328e 29560188 10.1039/c4sc02328e PMC5811097

[18] Kimura, H. (2013). Histone modifications for human epigenome analysis. *Journal of Human Genetics,**58*(7), 439–445. 10.1038/jhg.2013.66 23739122 10.1038/jhg.2013.66

[19] Farhadova, S., Gomez-Velazquez, M., & Feil, R. (2019). Stability and lability of parental methylation imprints in development and disease. *Genes,**10*(12), Article 999. 10.3390/genes10120999 31810366 10.3390/genes10120999 PMC6947649

[20] Cheedipudi, S., Genolet, O., & Dobreva, G. (2014). Epigenetic inheritance of cell fates during embryonic development. *Frontiers in Genetics,**5*, Article 19. 10.3389/fgene.2014.00019 24550937 10.3389/fgene.2014.00019 PMC3912789

[21] Song, K. A., & Faber, A. C. (2019). Epithelial-to-mesenchymal transition and drug resistance: Transitioning away from death. *Journal of Thoracic Disease,**11*(6), E82–E85. 10.21037/jtd.2019.06.11 31372302 10.21037/jtd.2019.06.11 PMC6626797

[22] Celià-Terrassa, T., & Kang, Y. (2016). Distinctive properties of metastasis-initiating cells. *Genes & Development,**30*(8), 892–908. 10.1101/gad.277681.116 27083997 10.1101/gad.277681.116 PMC4840296

[23] Bolis, M., Bossi, D., Vallerga, A., Ceserani, V., Cavalli, M., Impellizzieri, D., Di Rito, L., Zoni, E., Mosole, S., Elia, A. R., Rinaldi, A., Pereira Mestre, R., D'Antonio, E., Ferrari, M., Stoffel, F., Jermini, F., Gillessen, S., Bubendorf, L., Schraml, P., Calcinotto, A., … Theurillat, J. P. (2021). Dynamic prostate cancer transcriptome analysis delineates the trajectory to disease progression. *Nature communications, 12*(1), 7033. 10.1038/s41467-021-26840-5 10.1038/s41467-021-26840-5 PMC8640014 34857732

[24] Bai, Y., Zhang, Z., Cheng, L., Wang, R., Chen, X., Kong, Y., Feng, F., Ahmad, N., Li, L., & Liu, X. (2019). Inhibition of enhancer of zeste homolog 2 [EZH2] overcomes enzalutamide resistance in castration-resistant prostate cancer. *The Journal of Biological Chemistry,**294*(25), 9911–9923. 10.1074/jbc.RA119.008152 31085587 10.1074/jbc.RA119.008152 PMC6597805

[25] Venkadakrishnan, V. B., Presser, A. G., Singh, R., Booker, M. A., Traphagen, N. A., Weng, K., Voss, N. C., Mahadevan, N. R., Mizuno, K., Puca, L., Idahor, O., Ku, S. Y., Bakht, M. K., Borah, A. A., Herbert, Z. T., Tolstorukov, M. Y., Barbie, D. A., Rickman, D. S., Brown, M., & Beltran, H. (2024). Lineage-specific canonical and non-canonical activity of EZH2 in advanced prostate cancer subtypes. *Research Square*, rs.3.rs-3935288. 10.21203/rs.3.rs-3935288/v2 10.1038/s41467-024-51156-5 PMC11310309 39117665

[26] Zoma, M., Curti, L., Shinde, D., Albino, D., Mitra, A., Sgrignani, J., Mapelli, S. N., Sandrini, G., Civenni, G., Merulla, J., Chiorino, G., Kunderfranco, P., Cacciatore, A., Kokanovic, A., Rinaldi, A., Cavalli, A., Catapano, C. V., & Carbone, G. M. (2021). EZH2-induced lysine K362 methylation enhances TMPRSS2-ERG oncogenic activity in prostate cancer. *Nature Communications,**12*(1), 4147. 10.1038/s41467-021-24380-6 34230470 10.1038/s41467-021-24380-6 PMC8260656

[27] Entezari, M., Taheriazam, A., Paskeh, M. D. A., Sabouni, E., Zandieh, M. A., Aboutalebi, M., Kakavand, A., Rezaei, S., Hejazi, E. S., Saebfar, H., Salimimoghadam, S., Mirzaei, S., Hashemi, M., & Samarghandian, S. (2023). The pharmacological and biological importance of EZH2 signaling in lung cancer. *Biomedicine & Pharmacotherapy,**160*, Article 114313. 10.1016/j.biopha.2023.114313 36738498 10.1016/j.biopha.2023.114313

[28] O'Donnell, E., Muñoz, M., Davis, R., Randall, R. L., Tepper, C., & Carr-Ascher, J. (2024). Genetic and epigenetic characterization of sarcoma stem cells across subtypes identifies EZH2 as a therapeutic target. *bioRxiv: The preprint server for biology*, 2024.05.14.594060. 10.1101/2024.05.14.594060 10.1038/s41698-024-00776-7 PMC11717953 39789291

[29] Shen, J. K., Cote, G. M., Gao, Y., Choy, E., Mankin, H. J., Hornicek, F. J., & Duan, Z. (2016). Targeting EZH2-mediated methylation of H3K27 inhibits proliferation and migration of synovial sarcoma *in vitro*. *Scientific Reports,**6*, Article 25239. 10.1038/srep25239 27125524 10.1038/srep25239 PMC4850444

[30] Mieczkowska, I. K., Pantelaiou-Prokaki, G., Prokakis, E., Schmidt, G. E., Müller-Kirschbaum, L. C., Werner, M., Sen, M., Velychko, T., Jannasch, K., Dullin, C., Napp, J., Pantel, K., Wikman, H., Wiese, M., Kramm, C. M., Alves, F., & Wegwitz, F. (2021). Decreased PRC2 activity supports the survival of basal-like breast cancer cells to cytotoxic treatments. *Cell Death & Disease,**12*(12), 1118. 10.1038/s41419-021-04407-y 34845197 10.1038/s41419-021-04407-y PMC8630036

[31] Chen, H., Yu, S., Ma, R., Deng, L., Yi, Y., Niu, M., Xu, C., & Xiao, Z. J. (2024). Hypoxia-activated XBP1s recruits HDAC2-EZH2 to engage epigenetic suppression of ΔNp63α expression and promote breast cancer metastasis independent of HIF1α. *Cell Death and Differentiation,**31*(4), 447–459. 10.1038/s41418-024-01271-z 38413797 10.1038/s41418-024-01271-z PMC11043437

[32] Milazzo, G., Mercatelli, D., Di Muzio, G., Triboli, L., De Rosa, P., Perini, G., & Giorgi, F. M. (2020). Histone deacetylases [HDACs]: Evolution, specificity, role in transcriptional complexes, and pharmacological actionability. *Genes,**11*(5), Article 556. 10.3390/genes11050556 32429325 10.3390/genes11050556 PMC7288346

[33] Wang, B., Shen, X. Y., Pan, L. Y., Li, Z., Chen, C. J., Yao, Y. S., Tang, D. F., & Gao, W. (2023). The HDAC2-MTA3 interaction induces nonsmall cell lung cancer cell migration and invasion by targeting c-Myc and cyclin D1. *Molecular Carcinogenesis,**62*(11), 1630–1644. 10.1002/mc.23604 37401867 10.1002/mc.23604

[34] Karati, D., Mukherjee, S., & Roy, S. (2024). Emerging therapeutic strategies in cancer therapy by HDAC inhibition as a chemotherapeutic potent and epigenetic regulator. *Medical oncology [Northwood, London, England],**41*(4), 84. 10.1007/s12032-024-02303-x 38438564 10.1007/s12032-024-02303-x

[35] Bannister, A. J., & Kouzarides, T. (2011). Regulation of chromatin by histone modifications. *Cell Research,**21*(3), 381–395. 10.1038/cr.2011.22 21321607 10.1038/cr.2011.22 PMC3193420

[36] Tulsyan, S., Aftab, M., Sisodiya, S., Khan, A., Chikara, A., Tanwar, P., & Hussain, S. (2022). Molecular basis of epigenetic regulation in cancer diagnosis and treatment. *Frontiers in Genetics,**13*, Article 885635. 10.3389/fgene.2022.885635 36092905 10.3389/fgene.2022.885635 PMC9449878

[37] Seto, E., & Yoshida, M. (2014). Erasers of histone acetylation: The histone deacetylase enzymes. *Cold Spring Harbor Perspectives in Biology,**6*(4), Article a018713. 10.1101/cshperspect.a018713 24691964 10.1101/cshperspect.a018713 PMC3970420

[38] Park, S. Y., & Kim, J. S. (2020). A short guide to histone deacetylases including recent progress on class II enzymes. *Experimental & molecular medicine,**52*(2), 204–212. 10.1038/s12276-020-0382-4 32071378 10.1038/s12276-020-0382-4 PMC7062823

[39] Barneda-Zahonero, B., & Parra, M. (2012). Histone deacetylases and cancer. *Molecular Oncology,**6*(6), 579–589. 10.1016/j.molonc.2012.07.003 22963873 10.1016/j.molonc.2012.07.003 PMC5528343

[40] Brown, L. J., Achinger-Kawecka, J., Portman, N., Clark, S., Stirzaker, C., & Lim, E. (2022). Epigenetic therapies and biomarkers in breast cancer. *Cancers,**14*(3), Article 474. 10.3390/cancers14030474 35158742 10.3390/cancers14030474 PMC8833457

[41] Chianese, A., Santella, B., Ambrosino, A., Stelitano, D., Rinaldi, L., Galdiero, M., Zannella, C., & Franci, G. (2021). Oncolytic viruses in combination therapeutic approaches with epigenetic modulators: Past, present, and future perspectives. *Cancers,**13*(11), Article 2761. 10.3390/cancers13112761 34199429 10.3390/cancers13112761 PMC8199618

[42] Schech, A., Kazi, A., Yu, S., Shah, P., & Sabnis, G. (2015). Histone deacetylase inhibitor entinostat inhibits tumor-initiating cells in triple-negative breast cancer cells. *Molecular Cancer Therapeutics,**14*(8), 1848–1857. 10.1158/1535-7163.MCT-14-0778 26037781 10.1158/1535-7163.MCT-14-0778

[43] Shah, P., Gau, Y., & Sabnis, G. (2014). Histone deacetylase inhibitor entinostat reverses epithelial to mesenchymal transition of breast cancer cells by reversing the repression of E-cadherin. *Breast Cancer Research and Treatment,**143*(1), 99–111. 10.1007/s10549-013-2784-7 24305977 10.1007/s10549-013-2784-7

[44] Mann, B. S., Johnson, J. R., Cohen, M. H., Justice, R., & Pazdur, R. (2007). FDA approval summary: Vorinostat for treatment of advanced primary cutaneous T-cell lymphoma. *The Oncologist,**12*(10), 1247–1252. 10.1634/theoncologist.12-10-1247 17962618 10.1634/theoncologist.12-10-1247

[45] Wawruszak, A., Borkiewicz, L., Okon, E., Kukula-Koch, W., Afshan, S., & Halasa, M. (2021). Vorinostat [SAHA] and breast cancer: An overview. *Cancers,**13*(18), Article 4700. 10.3390/cancers13184700 34572928 10.3390/cancers13184700 PMC8468501

[46] Nouri Emamzadeh, F., Word, B., Cotton, E., Hawkins, A., Littlejohn, K., Moore, R., Miranda-Carbon, G., Orish, C. N., & Lyn-Cook, B. (2020). Modulation of estrogen α and progesterone receptors in triple negative breast cancer cell lines: The effects of Vorinostat and Indole-3-Carbinol in vitro. *Anticancer research, 40*(7), 3669–3683. 10.21873/anticanres.14356 10.21873/anticanres.14356 32620606

[47] Feng, J., Zhang, S., Wu, K., Wang, B., Wong, J. Y., Jiang, H., Xu, R., Ying, L., Huang, H., Zheng, X., Chen, X., & Ma, S. (2016). Combined effects of suberoylanilide hydroxamic acid and cisplatin on radiation sensitivity and cancer cell invasion in non-small cell lung cancer. *Molecular Cancer Therapeutics,**15*(5), 842–853. 10.1158/1535-7163.MCT-15-0445 26839308 10.1158/1535-7163.MCT-15-0445

[48] Pan, C. H., Chang, Y. F., Lee, M. S., Wen, B. C., Ko, J. C., Liang, S. K., & Liang, M. C. (2016). Vorinostat enhances the cisplatin-mediated anticancer effects in small cell lung cancer cells. *Bmc Cancer,**16*(1), 857. 10.1186/s12885-016-2888-7 27821078 10.1186/s12885-016-2888-7 PMC5100277

[49] Konstantinopoulos, P. A., Vandoros, G. P., & Papavassiliou, A. G. (2006). FK228 [depsipeptide]: A HDAC inhibitor with pleiotropic antitumor activities. *Cancer Chemotherapy and Pharmacology,**58*(5), 711–715. 10.1007/s00280-005-0182-5 16435156 10.1007/s00280-005-0182-5

[50] Yu, X. D., Wang, S. Y., Chen, G. A., Hou, C. M., Zhao, M., Hong, J. A., Nguyen, D. M., & Schrump, D. S. (2007). Apoptosis induced by depsipeptide FK228 coincides with inhibition of survival signaling in lung cancer cells. *Cancer journal,**13*(2), 105–113. 10.1097/PPO.0b013e318046eedc 17476138 10.1097/PPO.0b013e318046eedc

[51] Liu, W., Yu, L., Feng, Y., Huang, S., Hua, Y., Peng, M., Ruan, S., & Zhang, K. (2024). Which is more suitable for first-line treatment of extensive-stage small cell lung cancer, PD-L1 inhibitors versus PD-1 inhibitors? A systematic review and network meta-analysis. *The Clinical Respiratory Journal,**18*(7), Article e13804. 10.1111/crj.13804 39073269 10.1111/crj.13804 PMC11284309

[52] Kim, Y., Park, K., Kim, Y. J., Shin, S. W., Kim, Y. J., Choi, C., & Noh, J. M. (2022). Immunomodulation of HDAC inhibitor entinostat potentiates the anticancer effects of radiation and PD-1 blockade in the murine Lewis lung carcinoma model. *International Journal of Molecular Sciences,**23*(24), Article 15539. 10.3390/ijms232415539 36555180 10.3390/ijms232415539 PMC9779092

[53] Kim, Y. D., Park, S. M., Ha, H. C., Lee, A. R., Won, H., Cha, H., Cho, S., & Cho, J. M. (2020). HDAC inhibitor, CG-745, enhances the anti-cancer effect of anti-PD-1 immune checkpoint inhibitor by modulation of the immune microenvironment. *Journal of Cancer,**11*(14), 4059–4072. 10.7150/jca.44622 32368288 10.7150/jca.44622 PMC7196255

[54] Clermont, P. L., Lin, D., Crea, F., Wu, R., Xue, H., Wang, Y., Thu, K. L., Lam, W. L., Collins, C. C., Wang, Y., & Helgason, C. D. (2015). Polycomb-mediated silencing in neuroendocrine prostate cancer. *Clinical Epigenetics,**7*(1), 40. 10.1186/s13148-015-0074-4 25859291 10.1186/s13148-015-0074-4 PMC4391120

[55] Cheng, Y., Song, Z., Fang, X., & Tang, Z. (2024). Polycomb repressive complex 2 and its core component EZH2: Potential targeted therapeutic strategies for head and neck squamous cell carcinoma. *Clinical Epigenetics,**16*(1), 54. 10.1186/s13148-024-01666-2 38600608 10.1186/s13148-024-01666-2 PMC11007890

[56] Davies, A., Nouruzi, S., Ganguli, D., Namekawa, T., Thaper, D., Linder, S., Karaoğlanoğlu, F., Omur, M. E., Kim, S., Kobelev, M., Kumar, S., Sivak, O., Bostock, C., Bishop, J., Hoogstraat, M., Talal, A., Stelloo, S., van der Poel, H., Bergman, A. M., … Zoubeidi, A. (2021). An androgen receptor switch underlies lineage infidelity in treatment-resistant prostate cancer. *Nature Cell Biology,**23*(9), 1023–1034. 10.1038/s41556-021-00743-5 34489572 10.1038/s41556-021-00743-5 PMC9012003

[57] Kim, K. H., & Roberts, C. W. (2016). Targeting EZH2 in cancer. *Nature Medicine,**22*(2), 128–134. 10.1038/nm.4036 26845405 10.1038/nm.4036 PMC4918227

[58] Okasho, K., Mizuno, K., Fukui, T., Lin, Y. Y., Kamiyama, Y., Sunada, T., Li, X., Kimura, H., Sumiyoshi, T., Goto, T., Kobayashi, T., Lin, D., Wang, Y., Collins, C. C., Inoue, T., Ogawa, O., & Akamatsu, S. (2021). Establishment and characterization of a novel treatment-related neuroendocrine prostate cancer cell line KUCaP13. *Cancer Science,**112*(7), 2781–2791. 10.1111/cas.14935 33960594 10.1111/cas.14935 PMC8253279

[59] Fischetti, I., Botti, L., Sulsenti, R., Cancila, V., Enriquez, C., Ferri, R., Bregni, M., Crivelli, F., Tripodo, C., Colombo, M. P., & Jachetti, E. (2024). Combined therapy targeting AR and EZH2 curbs castration-resistant prostate cancer, enhancing anti-tumor T-cell response. *Epigenomics,**16*(9), 653–670. 10.2217/epi-2023-0374 38530086 10.2217/epi-2023-0374 PMC11160446

[60] Alves-Fernandes, D. K., & Jasiulionis, M. G. (2019). The role of SIRT1 on DNA damage response and epigenetic alterations in cancer. *International Journal of Molecular Sciences,**20*(13), Article 3153. 10.3390/ijms20133153 31261609 10.3390/ijms20133153 PMC6651129

[61] Latarani, M., Pucci, P., Eccleston, M., Manzo, M., Gangadharannambiar, P., Alborelli, I., Mongiardini, V., Mahmood, N., Colombo, M. P., Grimaldi, B., Rigas, S., Akamatsu, S., Hawkes, C., Wang, Y., Jachetti, E., & Crea, F. [2024]. EHZ2 inhibition enhances the activity of platinum chemotherapy in aggressive-variant prostate cancer. 10.1101/2024.09.06.611612

[62] Schade, A. E., Kuzmickas, R., Rodriguez, C. L., Mattioli, K., Enos, M., Gardner, A., & Cichowski, K. (2023). Combating castration-resistant prostate cancer by co-targeting the epigenetic regulators EZH2 and HDAC. *PLoS Biology,**21*(4), Article e3002038. 10.1371/journal.pbio.3002038 37104245 10.1371/journal.pbio.3002038 PMC10138213

[63] Liu, Q., Wang, G., Li, Q., Jiang, W., Kim, J. S., Wang, R., Zhu, S., Wang, X., Yan, L., Yi, Y., Zhang, L., Meng, Q., Li, C., Zhao, D., Qiao, Y., Li, Y., Gursel, D. B., Chinnaiyan, A. M., Chen, K., & Cao, Q. (2019). Polycomb group proteins EZH2 and EED directly regulate androgen receptor in advanced prostate cancer. *International Journal of Cancer,**145*(2), 415–426. 10.1002/ijc.32118 30628724 10.1002/ijc.32118 PMC7423571

[64] Kouznetsova, V. L., Tchekanov, A., Li, X., Yan, X., & Tsigelny, I. F. (2019). Polycomb repressive 2 complex-molecular mechanisms of function. *Protein Science,**28*(8), 1387–1399. 10.1002/pro.3647 31095801 10.1002/pro.3647 PMC6635771

[65] Margueron, R., & Reinberg, D. (2011). The polycomb complex PRC2 and its mark in life. *Nature,**469*(7330), 343–349. 10.1038/nature09784 21248841 10.1038/nature09784 PMC3760771

[66] Zhao, Y., Guan, Y. Y., Zhao, F., Yu, T., Zhang, S. J., Zhang, Y. Z., Duan, Y. C., & Zhou, X. L. (2022). Recent strategies targeting embryonic ectoderm development [EED] for cancer therapy: Allosteric inhibitors, PPI inhibitors, and PROTACs. *European Journal of Medicinal Chemistry,**231*, Article 114144. 10.1016/j.ejmech.2022.114144 35093670 10.1016/j.ejmech.2022.114144

[67] Tomassi, S., Romanelli, A., Zwergel, C., Valente, S., & Mai, A. (2021). Polycomb repressive complex 2 modulation through the development of EZH2-EED interaction inhibitors and EED binders. *Journal Of Medicinal Chemistry,**64*(16), 11774–11797. 10.1021/acs.jmedchem.1c00226 34351144 10.1021/acs.jmedchem.1c00226 PMC8404197

[68] Kim, W., Bird, G. H., Neff, T., Guo, G., Kerenyi, M. A., Walensky, L. D., & Orkin, S. H. (2013). Targeted disruption of the EZH2-EED complex inhibits EZH2-dependent cancer. *Nature Chemical Biology,**9*(10), 643–650. 10.1038/nchembio.1331 23974116 10.1038/nchembio.1331 PMC3778130

[69] Kumaraswamy, A., Duan, Z., Flores, D., Zhang, C., Sehrawat, A., Hu, Y. M., Swaim, O. A., Rodansky, E., Storck, W. K., Kuleape, J. A., Bedi, K., Mannan, R., Wang, X. M., Udager, A., Ravikumar, V., Bankhead, A., 3rd., Coleman, I., Lee, J. K., Morrissey, C., … Alumkal, J. J. (2023). LSD1 promotes prostate cancer reprogramming by repressing TP53 signaling independently of its demethylase function. *JCI Insight,**8*(15), Article e167440. 10.1172/jci.insight.167440 37440313 10.1172/jci.insight.167440 PMC10445684

[70] Chianese, U., Papulino, C., Passaro, E., Evers, T. M., Babaei, M., Toraldo, A., De Marchi, T., Niméus, E., Carafa, V., Nicoletti, M. M., Del Gaudio, N., Iaccarino, N., Randazzo, A., Rotili, D., Mai, A., Cappabianca, S., Mashaghi, A., Ciardiello, F., Altucci, L., & Benedetti, R. (2022). Histone lysine demethylase inhibition reprograms prostate cancer metabolism and mechanics. *Molecular Metabolism,**64*, Article 101561. 10.1016/j.molmet.2022.101561 35944897 10.1016/j.molmet.2022.101561 PMC9403566

[71] Kim, H., & Ronai, Z. A. (2020). PRMT5 function and targeting in cancer. *Cell Stress,**4*(8), 199–215. 10.15698/cst2020.08.228 32743345 10.15698/cst2020.08.228 PMC7380451

[72] Stopa, N., Krebs, J. E., & Shechter, D. (2015). The PRMT5 arginine methyltransferase: Many roles in development, cancer and beyond. *Cellular and Molecular Life Sciences,**72*(11), 2041–2059. 10.1007/s00018-015-1847-9 25662273 10.1007/s00018-015-1847-9 PMC4430368

[73] Liu, F., Xu, Y., Lu, X., Hamard, P. J., Karl, D. L., Man, N., Mookhtiar, A. K., Martinez, C., Lossos, I. S., Sun, J., & Nimer, S. D. (2020). PRMT5-mediated histone arginine methylation antagonizes transcriptional repression by polycomb complex PRC2. *Nucleic Acids Research,**48*(6), 2956–2968. 10.1093/nar/gkaa065 32025719 10.1093/nar/gkaa065 PMC7102951

[74] Deng, X., Shao, G., Zhang, H. T., Li, C., Zhang, D., Cheng, L., Elzey, B. D., Pili, R., Ratliff, T. L., Huang, J., & Hu, C. D. (2017). Protein arginine methyltransferase 5 functions as an epigenetic activator of the androgen receptor to promote prostate cancer cell growth. *Oncogene,**36*(9), 1223–1231. 10.1038/onc.2016.287 27546619 10.1038/onc.2016.287 PMC5322258

[75] He, Q., Zhang, Y., Li, W., Chen, S., Xiong, J., Zhao, R., Yuan, K., Hu, Q., Liu, S., Gao, G., Bedford, M. T., Tang, D. G., Xu, B., Zou, C., & Zhang, D. (2024). Inhibition of PRMT5 moderately suppresses prostate cancer growth in vivo but enhances its response to immunotherapy. *Cancer Letters,**602*, Article 217214. 10.1016/j.canlet.2024.217214 39218291 10.1016/j.canlet.2024.217214

[76] Pawar, J. S., Al-Amin, M. Y., & Hu, C. D. (2023). JNJ-64619178 radiosensitizes and suppresses fractionated ionizing radiation-induced neuroendocrine differentiation [NED] in prostate cancer. *Frontiers in Oncology,**13*, Article 1126482. 10.3389/fonc.2023.1126482 36959798 10.3389/fonc.2023.1126482 PMC10028149

[77] Ciarapica, R., Carcarino, E., Adesso, L., De Salvo, M., Bracaglia, G., Leoncini, P. P., Dall’agnese, A., Verginelli, F., Milano, G. M., Boldrini, R., Inserra, A., Stifani, S., Screpanti, I., Marquez, V. E., Valente, S., Mai, A., Puri, P. L., Locatelli, F., Palacios, D., & Rota, R. (2014). Pharmacological inhibition of EZH2 as a promising differentiation therapy in embryonal RMS. *Bmc Cancer,**14*, Article 139. 10.1186/1471-2407-14-139 24575771 10.1186/1471-2407-14-139 PMC4016511

[78] Sun, R., Shen, J., Gao, Y., Zhou, Y., Yu, Z., Hornicek, F., Kan, Q., & Duan, Z. (2016). Overexpression of EZH2 is associated with the poor prognosis in osteosarcoma and function analysis indicates a therapeutic potential. *Oncotarget,**7*(25), 38333–38346. 10.18632/oncotarget.9518 27223261 10.18632/oncotarget.9518 PMC5122393

[79] Kailayangiri, S., Altvater, B., Lesch, S., Balbach, S., Göttlich, C., Kühnemundt, J., Mikesch, J. H., Schelhaas, S., Jamitzky, S., Meltzer, J., Farwick, N., Greune, L., Fluegge, M., Kerl, K., Lode, H. N., Siebert, N., Müller, I., Walles, H., Hartmann, W., & Rossig, C. (2019). Ezh2 inhibition in Ewing sarcoma upregulates G_D2_ expression for targeting with gene-modified T cells. *Molecular Therapy: The Journal of the American Society of Gene Therapy,**27*(5), 933–946. 10.1016/j.ymthe.2019.02.014 30879952 10.1016/j.ymthe.2019.02.014 PMC6520468

[80] O’Brien, E., Tse, C., Tracy, I., Reddin, I., Selfe, J., Gibson, J., Tapper, W., Pengelly, R. J., Gao, J., Aladowicz, E., Petts, G., Thway, K., Popov, S., Kelsey, A., Underwood, T. J., Shipley, J., & Walters, Z. S. (2023). Pharmacological EZH2 inhibition combined with retinoic acid treatment promotes differentiation and apoptosis in rhabdomyosarcoma cells. *Clinical Epigenetics,**15*(1), Article 167. 10.1186/s13148-023-01583-w 37858275 10.1186/s13148-023-01583-w PMC10588044

[81] Kim, K. H., Kim, W., Howard, T. P., Vazquez, F., Tsherniak, A., Wu, J. N., Wang, W., Haswell, J. R., Walensky, L. D., Hahn, W. C., Orkin, S. H., & Roberts, C. W. (2015). SWI/SNF-mutant cancers depend on catalytic and non-catalytic activity of EZH2. *Nature Medicine,**21*(12), 1491–1496. 10.1038/nm.3968 26552009 10.1038/nm.3968 PMC4886303

[82] Schaefer, I. M., & Hornick, J. L. (2021). SWI/SNF complex-deficient soft tissue neoplasms: An update. *Seminars in Diagnostic Patholog,**38*(3), 222–231. 10.1053/j.semdp.2020.05.005 10.1053/j.semdp.2020.05.005 PMC7993547 32646614

[83] Knutson, S. K., Warholic, N. M., Wigle, T. J., Klaus, C. R., Allain, C. J., Raimondi, A., Porter Scott, M., Chesworth, R., Moyer, M. P., Copeland, R. A., Richon, V. M., Pollock, R. M., Kuntz, K. W., & Keilhack, H. (2013). Durable tumor regression in genetically altered malignant rhabdoid tumors by inhibition of methyltransferase EZH2. *Proceedings of the National Academy of Sciences of the United States of America,**110*(19), 7922–7927. 10.1073/pnas.1303800110 23620515 10.1073/pnas.1303800110 PMC3651445

[84] Kazansky, Y., Cameron, D., Mueller, H. S., Demarest, P., Zaffaroni, N., Arrighetti, N., Zuco, V., Kuwahara, Y., Somwar, R., Ladanyi, M., Qu, R., de Stanchina, E., Dela Cruz, F. S., Kung, A. L., Gounder, M. M., & Kentsis, A. (2024). Overcoming clinical resistance to EZH2 inhibition using rational epigenetic combination therapy. *Cancer Discovery,**14*(6), 965–981. 10.1158/2159-8290.CD-23-0110 38315003 10.1158/2159-8290.CD-23-0110 PMC11147720

[85] Munster, P. N., Thurn, K. T., Thomas, S., Raha, P., Lacevic, M., Miller, A., Melisko, M., Ismail-Khan, R., Rugo, H., Moasser, M., & Minton, S. E. (2011). A phase II study of the histone deacetylase inhibitor vorinostat combined with tamoxifen for the treatment of patients with hormone therapy-resistant breast cancer. *British Journal of Cancer,**104*(12), 1828–1835. 10.1038/bjc.2011.156 21559012 10.1038/bjc.2011.156 PMC3111195

[86] Tu, Y., Hershman, D. L., Bhalla, K., Fiskus, W., Pellegrino, C. M., Andreopoulou, E., Makower, D., Kalinsky, K., Fehn, K., Fineberg, S., Negassa, A., Montgomery, L. L., Wiechmann, L. S., Alpaugh, R. K., Huang, M., & Sparano, J. A. (2014). A phase I-II study of the histone deacetylase inhibitor vorinostat plus sequential weekly paclitaxel and doxorubicin-cyclophosphamide in locally advanced breast cancer. *Breast Cancer Research and Treatment,**146*(1), 145–152. 10.1007/s10549-014-3008-5 24903226 10.1007/s10549-014-3008-5

[87] Ramalingam, S. S., Maitland, M. L., Frankel, P., Argiris, A. E., Koczywas, M., Gitlitz, B., Thomas, S., Espinoza-Delgado, I., Vokes, E. E., Gandara, D. R., & Belani, C. P. (2010). Carboplatin and paclitaxel in combination with either vorinostat or placebo for first-line therapy of advanced non-small-cell lung cancer. *Journal of Clinical Oncology,**28*(1), 56–62. 10.1200/JCO.2009.24.9094 19933908 10.1200/JCO.2009.24.9094 PMC2799233

[88] Morgans, A. K., Hutson, T. E., Guan, A. K., Garcia, D., Zhou, A., Drea, E. J., & Vogelzang, N. J. (2021). 587p clinical and cost impact of cabazitaxel versus [vs] a second androgen receptor targeted agent [ARTA] for patients [pts] with metastatic castration-resistant prostate cancer [mCRPC] previously treated with docetaxel and the alternative ARTA [abiraterone or enzalutamide]. *Annals Of Oncology,**32*, S636–S637. 10.1016/j.annonc.2021.08.1100

[89] Iwata, H., Nakamura, R., Masuda, N., Yamashita, T., Yamamoto, Y., Kobayashi, K., Tsurutani, J., Iwasa, T., Yonemori, K., Tamura, K., Aruga, T., Tokunaga, E., Kaneko, K., Lee, M. J., Yuno, A., Kawabata, A., Seike, T., Kaneda, A., Nishimura, Y., … Saji, S. (2023). Efficacy and exploratory biomarker analysis of entinostat plus exemestane in advanced or recurrent breast cancer: Phase II randomized controlled trial. *Japanese Journal of Clinical Oncology,**53*(1), 4–15. 10.1093/jjco/hyac166 36398439 10.1093/jjco/hyac166 PMC9825728

[90] B., Zhang, Q., Hu, X., Li, Q., Sun, T., Li, W., Ouyang, Q., Wang, J., Tong, Z., Yan, M., Li, H., Zeng, X., Shan, C., Wang, X., Yan, X., Zhang, J., Zhang, Y., Wang, J., Zhang, L., Lin, Y., … Shang, H. (2023). Entinostat, a class I selective histone deacetylase inhibitor, plus exemestane for Chinese patients with hormone receptor-positive advanced breast cancer: A multicenter, randomized, double-blind, placebo-controlled, phase 3 trial. *Acta pharmaceutica Sinica. B*, *13*(5), 2250–2258. 10.1016/j.apsb.2023.02.001 10.1016/j.apsb.2023.02.001 PMC10213795 37250148

[91] Connolly, R. M., Zhao, F., Miller, K. D., Lee, M. J., Piekarz, R. L., Smith, K. L., Brown-Glaberman, U. A., Winn, J. S., Faller, B. A., Onitilo, A. A., Burkard, M. E., Budd, G. T., Levine, E. G., Royce, M. E., Kaufman, P. A., Thomas, A., Trepel, J. B., Wolff, A. C., & Sparano, J. A. (2021). E2112: Randomized phase III trial of endocrine therapy plus entinostat or placebo in hormone receptor-positive advanced breast cancer. A trial of the ECOG-ACRIN Cancer Research Group. *Journal of Clinical Oncology,**39*(28), 3171–3181. 10.1200/JCO.21.00944 34357781 10.1200/JCO.21.00944 PMC8478386

[92] Peterson, L. M., Kurland, B. F., Yan, F., Jiresova, A. N., Gadi, V. K., Specht, J. M., Gralow, J. R., Schubert, E. K., Link, J. M., Krohn, K. A., Eary, J. F., Mankoff, D. A., & Linden, H. M. (2021). ^18^F-fluoroestradiol PET imaging in a phase II trial of vorinostat to restore endocrine sensitivity in ER+/HER2- metastatic breast cancer. *Journal of Nuclear Medicine,**62*(2), 184–190. 10.2967/jnumed.120.244459 32591490 10.2967/jnumed.120.244459 PMC9364869

[93] Goldstein, L. J., Zhao, F., Wang, M., Swaby, R. F., Sparano, J. A., Meropol, N. J., Bhalla, K. N., Pellegrino, C. M., Katherine Alpaugh, R., Falkson, C. I., Klein, P., & Sledge, G. W. (2017). A phase I/II dy of suberoylanilide hydroxamic acid [SAHA] in combination with trastuzumab [Herceptin] in patients with advanced metastatic and/or local chest wall recurrent HER2-amplified breast cancer: A trial of the ECOG-ACRIN Cancer Research Group [E1104]. *Breast cancer research and treatment,**165*(2), 375–382. 10.1007/s10549-017-4310-9 28623430 10.1007/s10549-017-4310-9 PMC5682621

[94] Luu, T. H., Morgan, R. J., Leong, L., Lim, D., McNamara, M., Portnow, J., Frankel, P., Smith, D. D., Doroshow, J. H., Wong, C., Aparicio, A., Gandara, D. R., & Somlo, G. (2008). A phase II trial of vorinostat [suberoylanilide hydroxamic acid] in metastatic breast cancer: A California Cancer Consortium study. *Clinical Cancer Research,**14*(21), 7138–7142. 10.1158/1078-0432.CCR-08-0122 18981013 10.1158/1078-0432.CCR-08-0122 PMC3543872

[95] Connolly, R. M., Li, H., Jankowitz, R. C., Zhang, Z., Rudek, M. A., Jeter, S. C., Slater, S. A., Powers, P., Wolff, A. C., Fetting, J. H., Brufsky, A., Piekarz, R., Ahuja, N., Laird, P. W., Shen, H., Weisenberger, D. J., Cope, L., Herman, J. G., Somlo, G., … Stearns, V. (2017). Combination epigenetic therapy in advanced breast cancer with 5-azacitidine and entinostat: A phase II National Cancer Institute/stand up to cancer study. *Clinical Cancer Research,**23*(11), 2691–2701. 10.1158/1078-0432.CCR-16-1729 27979916 10.1158/1078-0432.CCR-16-1729 PMC5457329

[96] Roussos Torres, E. T., Ho, W. J., Danilova, L., Tandurella, J. A., Leatherman, J., Rafie, C., Wang, C., Brufsky, A., LoRusso, P., Chung, V., Yuan, Y., Downs, M., O’Connor, A., Shin, S. M., Hernandez, A., Engle, E. L., Piekarz, R., Streicher, H., Talebi, Z., … Connolly, R. M. (2024). Entinostat, nivolumab and ipilimumab for women with advanced HER2-negative breast cancer: A phase Ib trial. *Nature cancer,**5*(6), 866–879. 10.1038/s43018-024-00729-w 38355777 10.1038/s43018-024-00729-w PMC11552660

[97] Traynor, A. M., Dubey, S., Eickhoff, J. C., Kolesar, J. M., Schell, K., Huie, M. S., Groteluschen, D. L., Marcotte, S. M., Hallahan, C. M., Weeks, H. R., Wilding, G., Espinoza-Delgado, I., & Schiller, J. H. (2009). Vorinostat [NSC# 701852] in patients with relapsed non-small cell lung cancer: A Wisconsin Oncology Network phase II study. *Journal of Thoracic Oncology,**4*(4), 522–526. 10.1097/jto.0b013e3181952478 19347984 10.1097/jto.0b013e3181952478 PMC3050710

[98] Hoang, T., Campbell, T. C., Zhang, C., Kim, K., Kolesar, J. M., Oettel, K. R., Blank, J. H., Robinson, E. G., Ahuja, H. G., Kirschling, R. J., Johnson, P. H., Huie, M. S., Wims, M. E., Larson, M. M., Hernan, H. R., & Traynor, A. M. (2014). Vorinostat and bortezomib as third-line therapy in patients with advanced non-small cell lung cancer: A Wisconsin Oncology Network phase II study. *Investigational New Drugs,**32*(1), 195–199. 10.1007/s10637-013-9980-5 23728919 10.1007/s10637-013-9980-5 PMC4310688

[99] Han, J. Y., Lee, S. H., Lee, G. K., Yun, T., Lee, Y. J., Hwang, K. H., Kim, J. Y., & Kim, H. T. (2015). Phase I/II study of gefitinib [Iressa[®]] and vorinostat [IVORI] in previously treated patients with advanced non-small cell lung cancer. *Cancer Chemotherapy and Pharmacology,**75*(3), 475–483. 10.1007/s00280-014-2664-9 25552401 10.1007/s00280-014-2664-9 PMC4341018

[100] Reguart, N., Rosell, R., Cardenal, F., Cardona, A. F., Isla, D., Palmero, R., Moran, T., Rolfo, C., Pallarès, M. C., Insa, A., Carcereny, E., Majem, M., De Castro, J., Queralt, C., Molina, M. A., & Taron, M. (2014). Phase I/II trial of vorinostat [SAHA] and erlotinib for non-small cell lung cancer [NSCLC] patients with epidermal growth factor receptor [EGFR] mutations after erlotinib progression. *Lung Cancer,**84*(2), 161–167. 10.1016/j.lungcan.2014.02.011 24636848 10.1016/j.lungcan.2014.02.011

[101] Gray, J. E., Saltos, A., Tanvetyanon, T., Haura, E. B., Creelan, B., Antonia, S. J., Shafique, M., Zheng, H., Dai, W., Saller, J. J., Chen, Z., Tchekmedyian, N., Goas, K., Thapa, R., Boyle, T. A., Chen, D. T., & Beg, A. A. (2019). Phase I/Ib study of pembrolizumab plus vorinostat in advanced/metastatic non-small cell lung cancer. *Clinical Cancer Research,**25*(22), 6623–6632. 10.1158/1078-0432.CCR-19-1305 31409616 10.1158/1078-0432.CCR-19-1305 PMC7234799

[102] Hellmann, M. D., Jänne, P. A., Opyrchal, M., Hafez, N., Raez, L. E., Gabrilovich, D. I., Wang, F., Trepel, J. B., Lee, M. J., Yuno, A., Lee, S., Brouwer, S., Sankoh, S., Wang, L., Tamang, D., Schmidt, E. V., Meyers, M. L., Ramalingam, S. S., Shum, E., & Ordentlich, P. (2021). Entinostat plus pembrolizumab in patients with metastatic NSCLC previously treated with anti-PD-[L]1 therapy. *Clinical Cancer Research,**27*(4), 1019–1028. 10.1158/1078-0432.CCR-20-3305 33203644 10.1158/1078-0432.CCR-20-3305 PMC7887114

[103] Sharma, M. R., Subbiah, V., Shapiro, G., Pal, S. K., Agarwal, N., Wentzel, K., Fang, B., Zhang, N., Schwickart, M., Wang, Z., Curran, D., & Patnaik, A. (2022). 481p a phase I first-in-human study of XL092 in patients [pts] with locally advanced or metastatic solid tumors: Results from dose-escalation of XL092 alone and in combination with atezolizumab. *Annals Of Oncology,**33*, S760. 10.1016/j.annonc.2022.07.609

[104] Calvo, M., Penkov, K., Spira, A. I., Candilejo, I. M., Shore, N. D., Zhang, T., Mellado-Gonzalez, B., Gordoa, T. A., Rodriguez, L.P.-A., Tarantolo, S. R., Soto, J. J., Alter, R. S., Andreu-Vieyra, C., Bowler, T., Maity, A. K., Hariharan, S., & Schweizer, M. T. (2023). A multi-center, open-label, randomized dose expansion study of PF-06821497, a potent and selective inhibitor of enhancer of zeste homolog 2 [EZH2], in patients with metastatic castration-resistant prostate cancer [mCRPC]. *Journal of Clinical Oncolog,**41*(6_suppl), TPS282–TPS282. 10.1200/jco.2023.41.6_suppl.tps282

[105] Battisti, N. M. L., Welch, C., DeBelder, M., Deanfield, J., Weston, C., Peake, M. D., Sweeting, M., Adlam, D., & Ring, A. (2021). 1831p prevalence of cardiovascular disease in patients diagnosed with six common curable malignancies: A Virtual Cardio-Oncology Research Institute [VICORI] national registry dataset analysis. *Annals of Oncology,**32*, S1239. 10.1016/j.annonc.2021.08.719

[106] Yap, T. A., Winter, J. N., Giulino-Roth, L., Longley, J., Lopez, J., Michot, J. M., Leonard, J. P., Ribrag, V., McCabe, M. T., Creasy, C. L., Stern, M., Pene Dumitrescu, T., Wang, X., Frey, S., Carver, J., Horner, T., Oh, C., Khaled, A., Dhar, A., & Johnson, P. W. M. (2019). Phase I study of the novel enhancer of Zeste Homolog 2 [EZH2] inhibitor GSK2816126 in patients with advanced hematologic and solid tumors. *Clinical Cancer Research,**25*(24), 7331–7339. 10.1158/1078-0432.CCR-18-4121 31471312 10.1158/1078-0432.CCR-18-4121 PMC7377921

[107] Noce, B., Di Bello, E., Fioravanti, R., & Mai, A. (2023). LSD1 inhibitors for cancer treatment: Focus on multi-target agents and compounds in clinical trials. *Frontiers in Pharmacology,**14*, Article 1120911. 10.3389/fphar.2023.1120911 36817147 10.3389/fphar.2023.1120911 PMC9932783

[108] Hollebecque, A., Salvagni, S., Plummer, R., Niccoli, P., Capdevila, J., Curigliano, G., Moreno, V., de Braud, F., de Villambrosia, S. G., Martin-Romano, P., Baudin, E., Arias, M., de Alvaro, J., Parra-Palau, J. L., Sánchez-Pérez, T., Aronchik, I., Filvaroff, E. H., Lamba, M., Nikolova, Z., & de Bono, J. S. (2022). Clinical activity of CC-90011, an oral, potent, and reversible LSD1 inhibitor, in advanced malignancies. *Cancer,**128*(17), 3185–3195. 10.1002/cncr.34366 35737639 10.1002/cncr.34366 PMC9540525

[109] Li, W. J., Huang, Y., Lin, Y. A., Zhang, B. D., Li, M. Y., Zou, Y. Q., Hu, G. S., He, Y. H., Yang, J. J., Xie, B. L., Huang, H. H., Deng, X., & Liu, W. (2023). Targeting PRMT1-mediated SRSF1 methylation to suppress oncogenic exon inclusion events and breast tumorigenesis. *Cell reports,**42*(11), Article 113385. 10.1016/j.celrep.2023 37938975 10.1016/j.celrep.2023.113385

[110] Gounder, M., Schöffski, P., Jones, R. L., Agulnik, M., Cote, G. M., Villalobos, V. M., Attia, S., Chugh, R., Chen, T. W., Jahan, T., Loggers, E. T., Gupta, A., Italiano, A., Demetri, G. D., Ratan, R., Davis, L. E., Mir, O., Dileo, P., Van Tine, B. A., … Stacchiotti, S. (2020). Tazemetostat in advanced epithelioid sarcoma with loss of INI1/SMARCB1: An international, open-label, phase 2 basket study. *The Lancet. Oncology,**21*(11), 1423–1432. 10.1016/S1470-2045[20]30451-4 33035459 10.1016/S1470-2045(20)30451-4

[111] Jawad, M. U., Extein, J., Min, E. S., & Scully, S. P. (2009). Prognostic factors for survival in patients with epithelioid sarcoma: 441 cases from the SEER database. *Clinical Orthopaedics and Related Research,**467*(11), 2939–2948. 10.1007/s11999-009-0749-2 19224301 10.1007/s11999-009-0749-2 PMC2758965

[112] Dermawan, J. K., Singer, S., Tap, W. D., Nacev, B. A., Chi, P., Wexler, L. H., Ortiz, M. V., Gounder, M., & Antonescu, C. R. (2022). The genetic landscape of SMARCB1 alterations in SMARCB1-deficient spectrum of mesenchymal neoplasms. *Modern Pathology,**35*(12), 1900–1909. 10.1038/s41379-022-01148-x 36088476 10.1038/s41379-022-01148-x PMC9712236

[113] Gronchi, A., Miah, A. B., Dei Tos, A. P., Abecassis, N., Bajpai, J., Bauer, S., Biagini, R., Bielack, S., Blay, J. Y., Bolle, S., Bonvalot, S., Boukovinas, I., Bovee, J. V. M. G., Boye, K., Brennan, B., Brodowicz, T., Buonadonna, A., De Álava, E., Del Muro, X. G., Dufresne, A., … ESMO Guidelines Committee, EURACAN and GENTURIS. Electronic address: clinicalguidelines@esmo.org (2021). Soft tissue and visceral sarcomas: ESMO-EURACAN-GENTURIS Clinical Practice Guidelines for diagnosis, treatment and follow-up^☆^. *Annals of oncology: Official Journal of the European Society for Medical Oncology, 32*(11), 1348–1365. 10.1016/j.annonc.2021.07.006 10.1016/j.annonc.2021.07.006 34303806

[114] Hoy, S. M. (2020). Tazemetostat: First approval. *Drugs,**80*(5), 513–521. 10.1007/s40265-020-01288-x 32166598 10.1007/s40265-020-01288-x

[115] Chi, S. N., Yi, J. S., Williams, P. M., Roy-Chowdhuri, S., Patton, D. R., Coffey, B. D., Reid, J. M., Piao, J., Saguilig, L., Alonzo, T. A., Berg, S. L., Ramirez, N. C., Jaju, A., Mhlanga, J. C., Fox, E., Hawkins, D. S., Mooney, M. M., Takebe, N., Tricoli, J. V., … Parsons, D. W. (2023). Tazemetostat for tumors harboring SMARCB1/SMARCA4 or EZH2 alterations: Results from NCI-COG pediatric MATCH APEC1621C. *Journal of the National Cancer Institute,**115*(11), 1355–1363. 10.1093/jnci/djad085 37228094 10.1093/jnci/djad085 PMC11009504

[116] Constâncio, V., Nunes, S. P., Henrique, R., & Jerónimo, C. (2020). DNA methylation-based testing in liquid biopsies as detection and prognostic biomarkers for the four major cancer types. *Cells,**9*(3), Article 624. 10.3390/cells9030624 32150897 10.3390/cells9030624 PMC7140532

[117] Miller, J. L., & Grant, P. A. (2013). The role of DNA methylation and histone modifications in transcriptional regulation in humans. *Sub-Cellular Biochemistry,**61*, 289–317. 10.1007/978-94-007-4525-4_13 23150256 10.1007/978-94-007-4525-4_13 PMC6611551

[118] Van den Ackerveken, P., Lobbens, A., Pamart, D., Kotronoulas, A., Rommelaere, G., Eccleston, M., & Herzog, M. (2023). Epigenetic profiles of elevated cell free circulating H3.1 nucleosomes as potential biomarkers for non-Hodgkin lymphoma. *Scientific Reports,**13*(1), Article 16335. 10.1038/s41598-023-43520-0 37770512 10.1038/s41598-023-43520-0 PMC10539380

[119] Salani, F., Latarani, M., Casadei-Gardini, A., Gangadharannambiar, P., Fornaro, L., Vivaldi, C., Pecora, I., Massa, V., Marisi, G., Canale, M., Ulivi, P., Scartozzi, M., Eccleston, M., Masi, G., & Crea, F. (2022). Predictive significance of circulating histones in hepatocellular carcinoma patients treated with sorafenib. *Epigenomics,**14*(9), 507–517. 10.2217/epi-2021-0383 35473355 10.2217/epi-2021-0383

[120] Kusakabe, Y., Chiba, T., Oshima, M., Koide, S., Rizq, O., Aoyama, K., Ao, J., Kaneko, T., Kanzaki, H., Kanayama, K., Maeda, T., Saito, T., Nakagawa, R., Kobayashi, K., Kiyono, S., Nakamura, M., Ogasawara, S., Suzuki, E., Nakamoto, S., … Kato, N. (2021). EZH1/2 inhibition augments the anti-tumor effects of sorafenib in hepatocellular carcinoma. *Scientific Reports,**11*(1), Article 21396. 10.1038/s41598-021-00889-0 34725436 10.1038/s41598-021-00889-0 PMC8560765

[121] Italiano, A., Isambert, N., Metges, J.-P., Toulmonde, M., Cousin, S., Pernot, S., Spalato, M., Grellety, T., Auzanneau, C., Lortal, B., Kind, M., Le Loarer, F., Sellan-Albert, S., & Bellera, C. A. (2022). CAIRE: A basket multicenter open-label phase 2 study evaluating the EZH2 inhibitor tazemetostat in combination with durvalumab in patients with advanced solid tumors. *Journal of Clinical Oncology,**40*(16_suppl), TPS2703–TPS2703. 10.1200/jco.2022.40.16_suppl.tps2703

[122] Baca, S. C., Seo, J. H., Davidsohn, M. P., Fortunato, B., Semaan, K., Sotudian, S., Lakshminarayanan, G., Diossy, M., Qiu, X., El Zarif, T., Savignano, H., Canniff, J., Madueke, I., Saliby, R. M., Zhang, Z., Li, R., Jiang, Y., Taing, L., Awad, M., … Freedman, M. L. (2023). Liquid biopsy epigenomic profiling for cancer subtyping. *Nature Medicine,**29*(11), 2737–2741. 10.1038/s41591-023-02605-z 37865722 10.1038/s41591-023-02605-z PMC10695830

[123] Beltran, H., & Demichelis, F. (2021). Therapy considerations in neuroendocrine prostate cancer: What next? *Endocrine-Related Cancer,**28*(8), T67–T78. 10.1530/ERC-21-0140 34111024 10.1530/ERC-21-0140 PMC8289743

[124] Bayat Mokhtari, R., Homayouni, T. S., Baluch, N., Morgatskaya, E., Kumar, S., Das, B., & Yeger, H. (2017). Combination therapy in combating cancer. *Oncotarget,**8*(23), 38022–38043. 10.18632/oncotarget.16723 28410237 10.18632/oncotarget.16723 PMC5514969

[125] Frei, E., 3rd., Karon, M., Levin, R. H., Freireich, E. J., Taylor, R. J., Hananian, J., Selawry, O., Holland, J. F., Hoogstraten, B., Wolman, I. J., Abir, E., Sawitsky, A., Lee, S., Mills, S. D., Burgert, E. O., Jr., Spurr, C. L., Patterson, R. B., Ebaugh, F. G., James, G. W., 3rd., & Moon, J. H. (1965). The effectiveness of combinations of antileukemic agents in inducing and maintaining remission in children with acute leukemia. *Blood,**26*(5), 642–656. 5321112

[126] Jin, H., Wang, L., & Bernards, R. (2023). Rational combinations of targeted cancer therapies: Background, advances and challenges. *Nature Reviews. Drug Discovery,**22*(3), 213–234. 10.1038/s41573-022-00615-z 36509911 10.1038/s41573-022-00615-z

[127] Babar, Q., Saeed, A., Tabish, T. A., Pricl, S., Townley, H., & Thorat, N. (2022). Novel epigenetic therapeutic strategies and targets in cancer. *Biochimica Et Biophysica Acta. Molecular Basis Of Disease,**1868*(12), Article 166552. 10.1016/j.bbadis.2022.166552 36126898 10.1016/j.bbadis.2022.166552

[128] Lu, L., Zhan, M., Li, X. Y., Zhang, H., Dauphars, D. J., Jiang, J., Yin, H., Li, S. Y., Luo, S., Li, Y., & He, Y. W. (2022). Clinically approved combination immunotherapy: Current status, limitations, and future perspective. *Current Research in Immunology,**3*, 118–127. 10.1016/j.crimmu.2022.05.003 35676925 10.1016/j.crimmu.2022.05.003 PMC9167882

[129] Zhu, S., Zhang, T., Zheng, L., Liu, H., Song, W., Liu, D., Li, Z., & Pan, C. X. (2021). Combination strategies to maximize the benefits of cancer immunotherapy. *Journal of Hematology & Oncology,**14*(1), Article 156. 10.1186/s13045-021-01164-5 34579759 10.1186/s13045-021-01164-5 PMC8475356

[130] Fujiwara, Y., Mittra, A., Naqash, A. R., & Takebe, N. (2020). A review of mechanisms of resistance to immune checkpoint inhibitors and potential strategies for therapy. *Cancer drug resistance (Alhambra, Calif.),**3*(3), 252–275. 10.20517/cdr.2020.11 35582437 10.20517/cdr.2020.11 PMC8992481

[131] Mazzone, R., Zwergel, C., Mai, A., & Valente, S. (2017). Epi-drugs in combination with immunotherapy: A new avenue to improve anticancer efficacy. *Clinical Epigenetics,**9*, Article 59. 10.1186/s13148-017-0358-y 28572863 10.1186/s13148-017-0358-y PMC5450222

[132] Dunn, J., & Rao, S. (2017). Epigenetics and immunotherapy: The current state of play. *Molecular Immunology,**87*, 227–239. 10.1016/j.molimm.2017.04.012 28511092 10.1016/j.molimm.2017.04.012

[133] Kang, N., Eccleston, M., Clermont, P. L., Latarani, M., Male, D. K., Wang, Y., & Crea, F. (2020). EZH2 inhibition: A promising strategy to prevent cancer immune editing. *Epigenomics,**12*(16), 1457–1476. 10.2217/epi-2020-0186 32938196 10.2217/epi-2020-0186 PMC7607396

